# Remote consultations in sexual and reproductive health services: a systematic review of evidence on effectiveness, cost-effectiveness, experiences, access and equity

**DOI:** 10.1136/sextrans-2024-056458

**Published:** 2025-09-25

**Authors:** Charlotte Spurway, Iestyn Williams, Oluseyi Cyril Ayinde, Christian Bohm, Fiona Burns, Jo Gibbs, Jo Josh, Helen Munro, Melvina Woode Owusu, Danielle Solomon, Jonathan DC Ross, Louise J Jackson

**Affiliations:** 1 Health Economics Unit, School of Health Sciences, College of Medicine and Health, University of Birmingham, Birmingham, UK; 2 Health Services Management Centre, School of Social Policy and Society, University of Birmingham, Birmingham, UK; 3 Whittall Street Clinic, University Hospitals Birmingham NHS Foundation Trust, Birmingham, UK; 4 Institute for Global Health, University College London, London, UK; 5 Royal Free London NHS Foundation Trust, London, UK; 6 PPIE Lead, London, UK; 7 Hywel Dda University Health Board, Carmarthen, UK

**Keywords:** HEALTH SERVICES RESEARCH, POLICY, SEXUAL HEALTH, SYSTEMATIC REVIEW, CONTRACEPTION

## Abstract

**Abstract:**

**Objectives:**

Timely access to sexual health screening and contraception is an important public health issue. Substantial funding reductions for sexual and reproductive health services (SRHS) and COVID-19 have led to significant changes in service delivery, including the rapid introduction of remote consultations, as a substitute for in-person contact. There is limited evidence relating to the barriers to remote consultations and how these may impact sexual health outcomes and wider health inequalities. This study synthesises existing evidence on remote consultations to examine effectiveness, cost-effectiveness, experiences, access and equity.

**Methods:**

Eighteen electronic databases were systematically searched to locate relevant studies published from OECD (Organisation for Economic Co-operation and Development) countries after 2010. The Mixed Methods Appraisal Tool was used to evaluate the quality of the studies.

**Results:**

Out of 8690 studies identified, 48 met the inclusion criteria. The included studies were heterogeneous and covered a range of topics; however, few focused on health inequalities and remote consultations, with satisfaction and quality of care being the most common outcome measures. Many of the studies were completed post-2020 in response to COVID-19 and were of medium to low quality. Access to technology and communication challenges were found to impact inequality when accessing SRHS via remote consultations, although they enhance convenience for service users and service providers.

**Conclusions:**

Overall, the review shows that a range of studies have investigated remote consultations in SRHS, but there remains limited evidence on the impact on health inequalities and sexual health outcomes. The surge in post-2020 research, spurred by COVID-19, indicates the necessity for further high-quality research into equitable SRHS delivery in the post-COVID era, particularly in addressing technological and communication barriers. There is a need to further optimise access and the delivery of remote consultations in SRHS, considering the needs of service users, and minimising inequality (observed differences) and inequity (unfair differences).

**PROSPERO registration number:**

CRD42023397288.

**WHAT IS ALREADY KNOWN ON THIS TOPIC**

- Remote consultations in sexual and reproductive health services (SRHS) can increase convenience for users and may reduce provider costs.
- However, they may also create or worsen barriers to care, particularly for vulnerable populations.
- Current guidance on remote consultations in SRHS is based on limited evidence, regarding their impact on health inequalities, access, clinical effectiveness and cost-effectiveness, safeguarding and user acceptability.

**WHAT THIS STUDY ADDS**

- This systematic review identified several evidence gaps on remote consultations, including limited research on cost-effectiveness and a lack of long-term outcome data.
- This calls attention to the need for further research that examines ongoing impacts post-COVID-19.
- While remote consultations appear acceptable to both users and providers, important concerns were found relating to privacy and data protection and digital exclusion.
- More research is needed to assess and improve the equity of remote consultations.

**HOW THIS STUDY MIGHT AFFECT RESEARCH, PRACTICE OR POLICY**

- This study impacts research, practice and policy by highlighting gaps and areas for improvement in remote consultations within SRHS.
- It calls for more comprehensive research, particularly comparative and long-term studies, to better understand remote SRHS effectiveness and user experiences, especially among marginalised groups.
- The findings of this review suggest the need to update national SRHS guidelines to provide evidence-based support to service providers on how and when remote consultations should be used within services.

## Introduction

 Sexually transmitted infections (STIs) pose a significant global public health challenge, with the WHO identifying them as a priority.^1^ Young people, men who have sex with men (MSM), ethnic minorities and individuals from lower socioeconomic groups are particularly at risk.^2^ Additionally, challenges in accessing and using contraception persist in many parts of the world, especially in deprived areas.^3 4^ Access to sexual and reproductive health services (SRHS) is complex and influenced by various factors. The stigma associated with STIs and contraception creates barriers to access and, unlike many health services, most SRHS users self-refer.^5^ Timely attendance is important for rapidly identifying and treating STIs and preventing unplanned pregnancies.^6^

Remote consultations refer to the use of telecommunications or digital technology to replace in-person contact between clinicians and service users.^7^ Existing evidence suggests remote consultations can increase convenience for service users^8^ and potentially reduce costs for service providers,^9^ although evidence on cost-effectiveness is limited.^10 11^ There are concerns that remote consultations for SRHS could also create and/or exacerbate existing barriers to care and reduce choice for service users.^12^ This may disproportionately affect certain groups such as those from lower socioeconomic backgrounds, people from ethnically marginalised groups and individuals with mental health issues, worsening inequalities in experiences and outcomes.^12^ Digital exclusion is strongly associated with lower education and social disadvantage, increasing the risk of poorer outcomes for these individuals already at risk of poor sexual and reproductive health (SRH).^13^ Additional barriers also exist specifically for video consultations due to differing access to technology, connectivity and variation in digital literacy.^14^ Limited access to private spaces in the home can exacerbate existing inequities in access to SRHS.^15^

Current guidance is based on limited evidence on the role of remote consultations in SRHS, including the impact on health inequalities in relation to access, clinical effectiveness, cost-effectiveness, equity and experiences. It is important to identify evidence around which populations and conditions are most appropriate to manage remotely.^16^ The specific objectives of this review were to (1) identify literature relating to remote consultations in SRHS; (2) review the evidence on clinical effectiveness, cost-effectiveness, experience and access; (3) assess the evidence on impacts in relation to inequalities within the context of the UK and comparable countries.

## Methods

The protocol for the systematic review was registered in PROSPERO (CRD42023397288). The review followed the UK Centre for Review and Dissemination guidelines for systematic reviews in healthcare and PRISMA (Preferred Reporting Items for Systematic Reviews and Meta-Analyses) guidelines.

### Search strategy

Relevant published evidence was identified through databases from July 2023 to August 2023: MEDLINE, EMBASE, Health Management Information Consortium, PsycINFO, Cochrane Library, NHS Economic Evaluation Database (NHS EED), Web of Science-SCI, Applied Social Science Index and Abstracts (ASSIA), International Bibliography of the Social Sciences (IBSS), International Health Technology Assessment (HTA) database (INHTA), Database of Promoting Health Effectiveness Reviews (DoPHER), EconLit and Health Systems Evidence. A comprehensive search strategy was developed around remote consultations in SRHS in Organisation for Economic Co-operation and Development (OECD) countries using the PICO (population, intervention, comparator and outcomes) framework (online supplemental file 1). The review restricted the time period to 1 January 2011, when mobile phone use surpassed landline use,^17^ to August 2023.

- Population: Service users, healthcare professionals or other relevant stakeholders who have experience of or views about remote consultations related to SRHS including STI screening and treatment, contraception services, HIV and pre-exposure prophylaxis (PrEP).
- Intervention/interest: Evidence related to remote consultations, that is, video, phone, online or text message including evidence on ‘hybrid’ or flexible approaches, and those that focus on one modality.
- Comparator: Studies about remote consultations in SRHS were included regardless of the presence or absence of a comparison group. Where available, comparisons may include in-person consultations, outcomes or service utilisation.
- Outcomes: Clinical effectiveness, cost-effectiveness, experiences, access and equity.
- Exclusion criteria: Evidence was excluded if the population was under 16 years old, the focus was non-OECD countries or not related to SRHS.

### Screening

After deduplication, a two-stage process was used to screen the articles. In the first stage, abstracts and titles were reviewed by three reviewers (CS, LJJ, OCA). The second stage involved screening the full text of articles (CS, LJJ, IW) identified in the first stage. For a subset of articles, reviewers cross-checked each other’s decisions to ensure consistency and agreement in the inclusion and exclusion process, based on the criteria outlined in online supplemental file 2 and table 1.

**Table 1:** Summary characteristics of studies on remote consultations in SRHS

Author; country	Study aims and context	Remote consultation type	Consultation stage	Study scope				Study design	Target population*
				STI	CT	HIV	PrEP		
Aicken *et al*^56^; UK	Explore the acceptability of smartphone-enabled STI self-testing device to inform the development of a complex e-health intervention	Online care pathway	After positive STI test	**X**				Qualitative	Aged 16–24 years; sexually experienced; England
Aicken *et al*^62^; UK	Understand patient use and experiences of an e-health intervention	Online care pathway	Helpline for support	**X**				Qualitative	eSexual Health clinic users; age-group (16–24, ≥25 years)
Auchus *et al*^19^; USA	Examine whether telehealth appointments decrease non-attendance rates for HIV patients	Telehealth	HIV care retention			**X**	**X**	Cross-sectional	Patients at the University of California, San Francisco 360 Wellness Centre
Baraitser and Lupton^61^; UK	Evaluate user experience and clinical implications of the digitised diagnosis and management of genital herpes and warts	Photo-diagnosis service	Main appointment—diagnosis	**X**				Qualitative	Users of an online photo-diagnosis service; London
Beck *et al*^26^; Spain	Investigate the efficiency of the EmERGE pathway of care for medically stable people living with HIV in Spain	Video and mobile app	HIV treatment and monitoring			**X**	**X**	Before-and-after	People living with HIV in Barcelona; age ≥18 years
Beck *et al*^25^; UK	Estimate the efficiency of implementing the EmERGE‡ pathway of care for people living with medically stable HIV in England	Video and mobile app	HIV treatment and monitoring			**X**	**X**	Before-and-after	People living with HIV in Brighton; age ≥18 years
Bissessor *et al*^20^; Australia	Determine differences in HIV rate testing and dictation before and after telephone HIV results were introduced	Telephone	HIV result notification and discussions			**X**		Before and after	Men testing for HIV at Melbourne Sexual Health Centre
Bittleston *et al*^52^; Australia	Explore experiences of using telehealth to discuss SRH-related issues during 2020	Telehealth	All stages of care	**X**	**X**			Qualitative	Aged ≥18 years; and living in Australia
Bosó Pérez *et al*^58^; UK	Explore the challenges for patients accessing SRH services during the pandemic	Telemedicine	All stages of care	**X**	**X**	**X**	**X**	Qualitative	People reporting an unmet need for SRH services in the Natsal-COVID survey; UK
Bryson *et al*^24^; USA	Describe AYAs follow-up care for LARC via telemedicine in the year following COVID-19 onset	Telemedicine—Telephone and video	Follow-up		**X**			Longitudinal cohort	AYAs using LARC, aged 13–25 years; using Boston Children’s Hospital, Children’s Hospital Los Angeles, Seattle Children’s Hospital and Children’s Hospital at Montefiore
Clure *et al*^30^; US	Assess rural women’s preferences, experiences and perceived access to reproductive healthcare in rural communities	Telemedicine	All stages of care	**X**	**X**			Cross-sectional	Women aged 18–45 years; Rural Colorado
Comfort *et al*^22^; USA	Assess clinical practice change during COVID-19 and strategies adopted to maintain contraceptive care	Telemedicine, mail-order pharmacies, curbside delivery and self-administered injections	All stages of care		**X**			Cross-sectional	Contraception providers and clinic staff in USA
Conway *et al*^31^; Australia	Determine links between client characteristics, result receipt, HIV telephone result satisfaction and result preferences	Telephone	HIV result notification and discussions			**X**		Cross-sectional	Patients with low risk for HIV infection from two sexual health services in Sydney
Ennis *et al*^23^; USA	Examine factors associated with successful completion of video telehealth appointments for at risk or patients with HIV	Two-way video-audio connection	HIV treatment and monitoring			**X**	**X**	Cohort	Patients with or at risk of HIV; Florida;
Estcourt *et al*^28^; UK	Assess the safety and feasibility of an E-Sexual Health Clinic as an alternative to routine in-person care	Online clinical care pathway	Testing and follow-up	**X**				Exploratory proof-of-concept	People aged ≥16 years; untreated Chlamydia; from six areas of England
Galaviz *et al*^32^; USA	Explore patient experiences with telemedicine for HIV care during the first wave of COVID-19 in Southern United States	Telephone	HIV treatment and monitoring			**X**	**X**	Cross-sectional	Adults ≥18 years; receiving care from a clinic in Atlanta
Garrett *et al*^33^; Australia	Examine young adults’ pre-use views on webcam and telephone consultations for sexual health in Australia	Telephone and webcam	All stages of care	**X**				Cross-sectional	Aged 16–24 years; living in Australia
Giorlando *et al*^57^; USA	Investigate the acceptability and feasibility of using remote services to expand access to PrEP across Mississippi	Telemedicine—phone and/or video	Testing and PrEP delivery			**X**	**X**	Mixed methods	PrEP-eligible adults; North Jackson, Hattiesburg, Gulfport, and the Delta region of Mississippi
Gluskin *et al*^34^; USA	Examine perspectives and associations on SRH risk-reduction counselling via videoconference	Video	All stages of care	**X**				Cross-sectional	AFAB; aged 21–24 years; patient of a midwest family planning clinic
Golub *et al*^73^; USA	Develop novel strategy for expanding HIV partner services and examine the feasibility, acceptability and impact	Telephone	All stages of care	**X**		**X**	**X**	Feasibility and acceptability	Partners of disease intervention specialists index patients; New York City
Greenwell *et al*^27^; USA	Evaluate differences in outcomes between patients who received in-person PrEP care and those who received PrEP care via telehealth	Telehealth via telephone	PrEP prescription			**X**	**X**	Retrospective cohort	≥18 years; and had ≥2 PrEP prescriptions; Aurora, Colorado
Hill *et al*^50^; USA	Explore potential racial and ethnic disparities in adoption and use of telehealth services in family planning services	Telehealth	All stages of care	**X**	**X**			Cohort	AFAB patients; Arkansas, Kansas, Missouri and Oklahoma
Hoth *et al*^63^; USA	Describe programme results and share lessons for the implementation of PrEP telehealth programmes	Video	PrEP initiation and monitoring			**X**	**X**	Cohort	Individuals with need for PrEP; Iowa
Huang *et al*^42^; USA	Explore provider perspectives on the implementation of telehealth	Telephone and video	All stages of care		**X**			Qualitative	Providers of contraceptive care; Illinois
Johnson^60^; USA	Explore opinions about getting birth control prescriptions online	Telemedicine	All stages of care		**X**			Cross-sectional	Women under 60
Kavanaugh and Zolna^35^; USA	Examine where and how reproductive age women in three US states want to get their contraception	Telemedicine (over the computer or phone)	All stages of care		**X**			Cross-sectional	Women† aged 18–44 years; live in Arizona, New Jersey or Wisconsin
Lindberg *et al*^51^; USA	Identify characteristics of those using telehealth and examine respondents’ evaluation of the quality of care received	Telemedicine—telephone or video	All stages of care		**X**			Cross-sectional	AFAB; aged 18–49 years; and that ever had penile–vaginal sex; residing in a US household.
Lunt *et al*^36^; UK	Assess the attitudes of sexual healthcare professionals towards digitisation of SRHS in the UK at early COVID-19 stage	Telephone, email, social media, web chat or phone applications	All stages of care	**X**	**X**	**X**		Mixed methods	UK sexual healthcare professionals
Mastorino *et al*^49^; Italy	Evaluate performance and adequacy of nursing telephone for triaging symptomatic patients	Telephone	Triage	**X**				Observational cohort	Users of the Centre for Sexual Health of Turin
Merz-Herrala *et al*^64^; USA	Examine differences in access, telehealth and in-person visits and telehealth quality for contraceptive visits in the USA during the COVID-19 pandemic	Telephone, video or online/chat	All stages of care		**X**			Cross-sectional	Identify as a woman; aged 18–45 years; living in the USA
Nadarzynski *et al*^37^; UK	Assess the acceptability of video consultations, live webchats and chatbots among patients attending SRHS	Video consultations, live webchats and AI-enabled chatbots	All stages of care	**X**	**X**			Cross-sectional	Patients attending SRH clinic; Hampshire, UK
Phillips *et al*^21^; Australia	Determine the changes in service delivery to patient populations during the lockdown period between March and May 2020	Telephone	All stages of care	**X**	**X**	**X**		Cross-sectional	Directors of public sexual health clinics across Australia
Player *et al*^38^; USA	Evaluate the feasibility, acceptability and satisfaction of PrEP delivery via telehealth and assess medication adherence	Video visits	PrEP initiation and monitoring				**X**	Feasibility	HIV negative and eligible for PrEP patients; living in Southeastern USA
Rao *et al*^43^; USA	Capture provider’s voices and experiences using telehealth for contraception during COVID-19	Telehealth services	All stages of care		**X**			Secondary qualitative	Contraceptive providers across the USA
Refugio *et al*^39^; USA	Investigate the feasibility of a telehealth intervention to initiate and deliver PrEP	Telehealth visits through telephone	PrEP initiation and monitoring				**X**	Feasibility	HIV-uninfected YMSM; 18–25 years; from the San Francisco Bay Area
Rose *et al*^40^; New Zealand	Explore young people’s needs for sexual healthcare during lockdown, experiences of care and preferences for future care	Telehealth methods: telephone, video and messaging applications	Triage and consultation	**X**	**X**			Cross-sectional	Aged 15–24 years; from a high deprivation region of New Zealand
Ryu *et al*^44^; Canada	Investigate the impact of COVID-19 on testing from provider perspectives and the adoption of alternative models of sexual healthcare	Online-based STBBI testing service	All stages of care	**X**		**X**	**X**	Qualitative	Sexual health service providers in Ontario, Canada
Shanks *et al*^72^; UK	Assess the volume of SMS text messages and nature of messages sent by users	Two-way text messaging	SRH advice and support	**X**	**X**	**X**	**X**	Multi-methods	Users of SH:24, an online SRH provider in the UK
Stifani *et al*^48^; USA	Describe contraception provider’s experiences of telemedicine during COVID-19 and their preferences and recommendations for telemedicine	Billable clinical visits using technology (typically phone or video)	Contraception counselling—excluding follow-up		**X**			Mixed methods	Family planning providers practicing in the USA
Sullivan *et al*^59^; UK	Establish factors impacting online disclosure of safeguarding concerns and views about an appropriate response to an online disclosure	Online SRH services	Initiation	**X**				Qualitative	Young people aged 16–21 years; safeguarding experts
Sundstrom *et al*^54^; USA	Understand women’s contraceptive needs and perceptions of access through telehealth services in rural communities	Telehealth such as video conference or on the computer	All stages of care		**X**			Qualitative	Women; 18–44 years; living in five rural counties in South Carolina
Üsküp *et al*^45^; USA	Explore provider’s perspectives on the acceptability and appropriateness of digital technology to improve PrEP uptake and access for BLCW	Stand-alone telemedicine platforms, clinically integrated telemedicine platforms and texting service	PrEP uptake and initiation			**X**	**X**	Formative implementation research project	HIV service organisations in Los Angeles County
Wells *et al*^53^; Australia	Perspectives of receiving or giving positive HIV test results over the phone and providing HIV-related care	Telephone	Conveying HIV+results			**X**		Qualitative	People with recent HIV diagnosis; aged ≥16 years; HIV healthcare providers; Australia
Yarger *et al*^41^; USA	Examine perceived access to telemedicine for contraception by food and housing insecurity status	Telephone and video	All stages of care		**X**			Cross-sectional	AFAB; aged 16–25 years; had vaginal sex in last 12 months and not pregnant; attending community college in Texas or California
Yelverton *et al*^46^; USA	Understand telehealth utilisation and the barriers for HIV care services in South Carolina during COVID-19	Telehealth	All stages of care			**X**	**X**	Qualitative	Relevant HIV/AIDS stakeholders in South Carolina
Zapata *et al*^29^; USA	Investigate the impact of COVID-19 on family planning service provisions from the perspective of US physicians	Telehealth	All stages of care		**X**			Cross-sectional	Physicians providing family planning in the USA
Zhang *et al*^47^; USA	Assess the telemedicine experience in healthcare including PrEP from key stakeholders	Telemedicine (eg, telephone, computer and mobile phones)	All stages of care			**X**	**X**	Qualitative	Primary care providers practicing in New York state and PrEP eligible women; aged ≥18 years; reside in New York state
Zimbile *et al*^55^; Netherlands	Assess acceptance, care quality and evaluation of video vs in-person consultations	Video	All stages of care	**X**	**X**			Mixed methods	Young clients aged 15–25 years; nurses; 9 SRH clinics in the Netherlands

‘All stages of care’ include studies that did not specifically mention a stage of the consultation process.

* Target population includes age and location where this information is available. However, not all studies provide target sample age or further details. This is why some rows include age and specify a city or area, while others do not.

† Study specifically mentions the inclusion of transwomen and gender diverse individuals.

‡ *The EmERGE pathway was developed as part of the 'Evaluating mHealth technology in HIV to improve Empowerment and healthcare utilization: Research and Innovation to Generate Evidence for Personalized Care* (EmERGE) Project'

AFAB, assigned-female at birth; AI, artificial intelligence; AYAs, adolescents and young adults; BLCW, black Latina cisgender women; CT, contraception; LARC, long-acting reversible contraception; PrEP, pre-exposure prophylaxis; SRH, sexual and reproductive health; SRHS, sexual and reproductive health services; STBBI, sexually transmitted and blood-borne infection; STIs, sexually transmitted infections; YMSM, young men who have sex with men.

### Data extraction and quality assessment

Three reviewers (CS, LJJ, IW) completed data extraction using a standardised pre-piloted form tailored to the study’s objectives. The information extracted from each paper included background, consultation type and study scope, in addition to information about study aims, design and outcomes. To assess the quality of the evidence, the Mixed Methods Appraisal Tool (MMAT)^18^ was used to inform the analysis but not to exclude studies. Quality assessment was completed by one reviewer (CS) and was checked and confirmed by two additional reviewers (LJJ and IW).

### Synthesis

The synthesis aimed to provide an overview of current evidence and recommendations. The information and data from the evidence were collated and tabulated and then compared using a narrative approach. We also synthesised findings relating to the reported role of remote consultations in reducing and/or exacerbating health inequities according to a range of protected characteristics. The final step of the synthesis was to develop an understanding of the existing evidence on remote consultations in SRHS, the gaps in terms of equity and implications for practice and policy.

## Results

**Figure 1:**
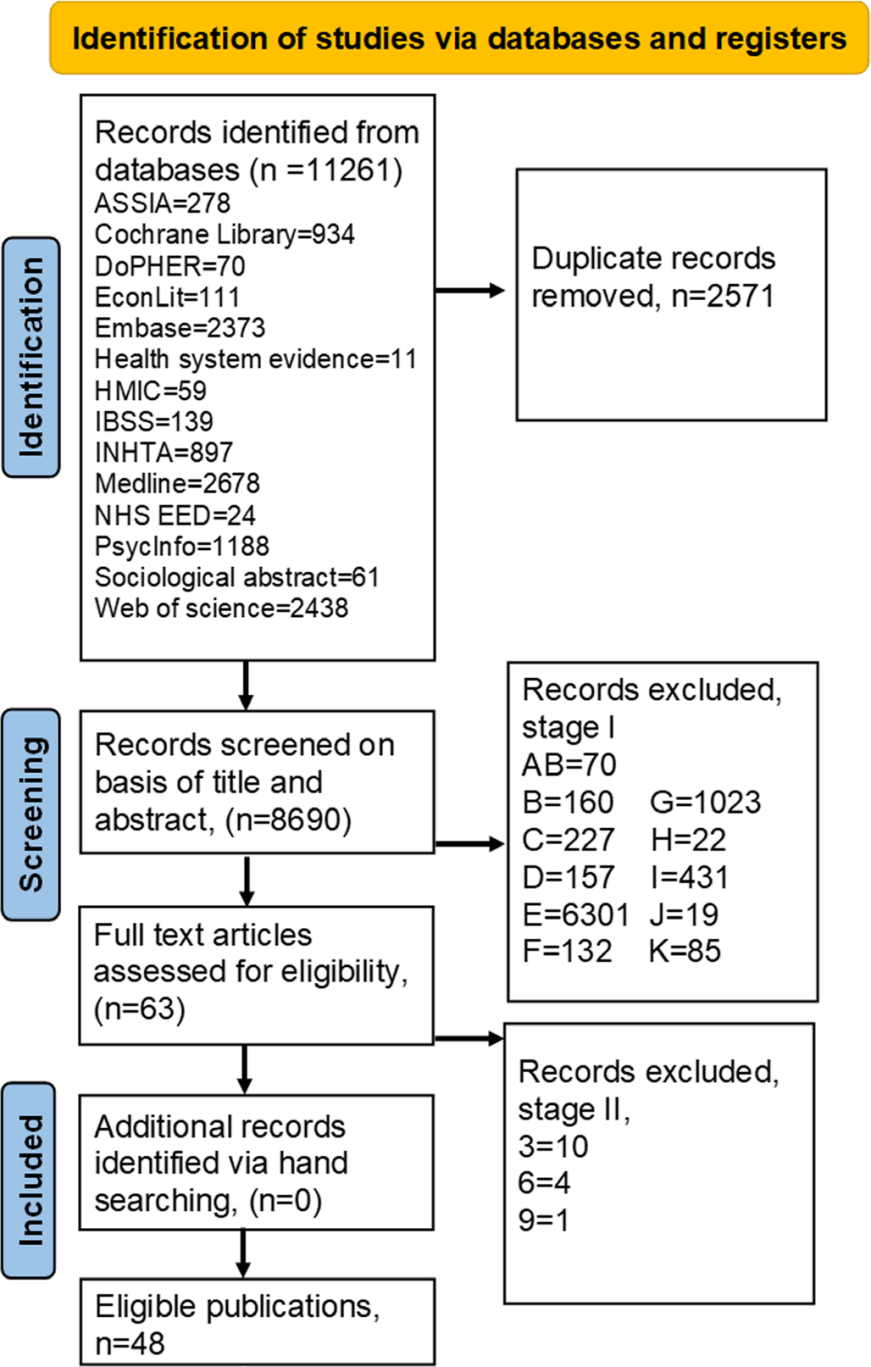
AB=Abortion studies. (*This systematic review also screened studies on abortion and pregnancy in relation to sexual and reproductive health services (SRHS) up to stage 2. However, these will be reviewed separately as the scope of the review became too broad.*) B=The study involves remote consultations; however, it is unclear if associated with SRHS. C=Unclear if the study falls under eligible criteria, but may be useful for the review. D=The study is concerned with consultations but not clear if relevant to remote consultations. E=The study is not relevant to remote consultations in SRHS. F=Copy/duplicate publication. G=Non-OECD (Organisation for Economic Co-operation and Development) publication. H=Publication predates 1 January 2011. I=No abstract. J=Abstract not published in English. K=No methods section. 3=Review, guideline or editorial in relation to remote consultation in SRHS. 6=Not relevant to remote consultation in SRHS. 9=Duplicate study. ASSIA, Applied Social Science Index and Abstracts; DoPHER, Database of Promoting Health Effectiveness Reviews; HMIC, Health Management Information Consortium; IBSS, International Bibliography of the Social Sciences; INHTA, International Health Technology Assessment (HTA) database; NHS EED, NHS Economic Evaluation Database.

From 11 261 articles identified, 2571 duplicates were excluded, leaving 8690 articles for title and abstract screening (figure 1). Among these, 48 studies were found to be eligible for inclusion.

### Study characteristics

Among the 48 studies, research aims, study design and outcome measures were varied, making direct comparisons difficult (table 1). Many studies did not clearly define remote consultations but used broad definitions, such as online clinical services, telehealth or telemedicine. There were 29 quantitative studies, 14 qualitative and 5 mixed-methods or multi-method. Study scopes varied, with 13 investigating a mixture of SRHS remits (eg, STIs, contraception, HIV/PrEP, safeguarding), 7 focused solely on STIs, 12 on contraception and 16 on HIV/PrEP. Most studies were conducted in the USA (n=27), followed by UK (n=10), Australia (n=6), and one each from Italy, New Zealand, Canada, Spain and the Netherlands. Studies covered various service providers, including SRH clinics, community services, outpatient services, online SRH providers and pharmacies.

Reflecting study scopes and designs, the population groups in the studies were also varied. 24 studies had women-only or women-majority samples, while 12 studies had men-only or male-majority samples; others did not report gender. 11 studies included gender-diverse individuals (eg, transgender or non-binary), although they represented a relatively small proportion of the sample. Most participants were over 25 years old, unless studies specifically recruited younger people, and age was not often reported in service provider studies. Information on racial or ethnic diversity was limited, with many studies not reporting ethnicity. Those that did had predominantly white samples (n=24), a few had black-majority samples (n=4) and one had an equal mix of white and black participants. Sexual identity was not always reported, though nine studies focused on MSM, lesbian or gay participants. More specific details are presented about the studies in table 2 (quantitative studies), table 3 (mixed methods studies) and table 4 (qualitative studies).

**Table 2:** Main findings for the quantitative studies on remote consultations in SRHS

Study	Sample size	Primary outcome measures	Comparator	Data source	Main findings
Beck *et al*^26^	546	Unit and annual costs pre- and post-EmERGE per patient-year (PPY); changes in CD4 count and viral load, patient activation and quality of life	None	Primary	CD4 counts did not differ significantly between periods. Patient activation and quality of life indicators did not change substantially over time. Total average annual costs increased from €8430 (95% CI €8356 to €8514) to €8515 (95% CI €8441 to €8595) PPY.
Beck *et al*^25^	565	Unit and annual costs pre- and post-EmERGE PPY; changes in CD4 count and viral load, patient activation and quality of life‡	None	Primary	CD4 counts did not differ significantly between periods. Patient activation and quality of life indicators did not change substantially over time. Total average annual costs for all visits decreased from £751 PPY (95% CI £722 to £780) pre-EmERGE to £678 PPY (95% CI £653 to £705) post-EmERGE.
Bissessor *et al*^20^	7280*	STI testing rate; mean time spent by men on an attendance; other clinical outcomes	In-person	Secondary	Modest increase of 3.2% (p<0.001) in HIV tests. Mean time spent in-person: 22.1 min; mean time spent telephone: 2.5 min. Telephone saved 516 hours of consultation time in 2013–2014.
Bryson *et al*^24^	319	Patient characteristics and visit information; patient-reported symptoms	Usual care	Secondary	63.4% of follow-ups were in person vs 36.6% via telemedicine. 62.7% occurred within 12 months. Reports of symptoms were not associated with mode of follow-up.
Clure *et al*^30^	478	Perceived difficulty accessing reproductive healthcare; interest in telemedicine	None	Primary	Most common barriers: too few community-based providers (81.4%) and long distance to care (69.5%). 51.0% had used telemedicine before and 52.5% were interested in using telemedicine.
Comfort *et al*^22^	907	Seeing new patients; offering contraceptive counselling; offering the full range of methods; offering care to marginalised patients	Clinic status	Primary	80% providers remained open during COVID-19. 11% offered telemedicine pre-pandemic and 79% offered during the pandemic.
Conway *et al*^31^	405	Satisfaction and preference of receipt of HIV results	None	Primary	97.1% were satisfied with the delivery of their HIV results by telephone. Email and text message for results were favoured by very few clients.
Ennis *et al*^23^	4873	Telehealth medical appointment completion	Visit completion	Secondary	Factors found to be negatively associated with video telehealth visit completion were black/African American (p<0.01), heterosexual (p<0.01), Hispanic/Latinx (p<0.02), public insurance (p<0.01), and detectable viral load (p<0.01).
Estcourt *et al*^28^	343†	Consent to the online chlamydia pathway and receipt of appropriate treatment	GUM clinics	Primary	In GUM and NCSP patients, 97% and 89% received treatment, with 74 and 60 patients treated online. Median time to collect treatment was 1 day for both.
Galaviz *et al*^32^	101	Quality, usefulness, satisfaction and concerns with telemedicine	None	Primary	Median telemedicine visits per patient=1 (avg. time=15 min). Median quality score: 6.5/7, median usefulness: 6.0/7, and median satisfaction: 6.3/7 with telemedicine. 28% were concerned about lack of examinations and clinical tests.
Garrett *et al*^33^	662	Willingness and preferences for consultations different media; views of webcam, telephone and in-person consultations.	In-person	Primary	Willingness: 85% in-person, 63% telephone and 29% webcam. Men and people with same-sex partners are more willing to have webcam consultations. Preference for remote appointments increased with increased travel time.
Gluskin *et al*^34^	120	Impact of COVID-19 on mood and healthcare access; SRH risk behaviours; opinions of virtual healthcare	None	Primary	COVID-19 impacted mood and access. Nearly all participants had SRH risk. 72.2% were comfortable with videoconference technology and 67.6% had sufficient privacy during videoconference.
Greenwell *et al*^27^	149	Tablets prescribed per patient-year; estimated medication possession; HIV screens and STI per patient-year; creatinine tests per patient-year; number of patients lost to follow-up	In-person	Secondary	In-person clinic: PrEP tablets filled per person-year was 324.4; telehealth clinic: 320.6. HIV screens per person-year were 3.6 in the in-person clinic, compared with 3.4 in the telehealth clinic. No HIV infections occurred across either clinic. Patients were less likely to be lost to follow-up with telehealth (p=0.009).
Hill *et al*^50^	3142	Uptake of new family planning telehealth services by race/ethnicity	In-person	Secondary	54.5% appointments were for contraception, 28.5% for STI-related concerns and 17.0% for gynaecological concerns. Most patients receiving in-clinic and telehealth were White (> 58%).
Hoth *et al*^63^	127	Referrals to TelePrEP by source; completion of initial video visits; Initiation of PrEP; retention in TelePrEP	None	Primary	TelePrEP received 186 referrals, 68% completed initial video visit with a pharmacist. No variation in completion rates by referral source (p=0.18). 91% started PrEP and retention at 6 months was 61%.
Johnson^60^	252	Likelihood to use online birth control services; comfort with online birth control services	None	Primary	Significant regression equations were found between positive attitudes towards direct-to-consumer advertising and telemedicine and comfort and likelihood to use telemedicine for contraception. Comfort mediates relationships between likelihood of telemedicine use and positive attitudes.
Kavanaugh and Zolna^35^	2804	Preferred contraception sources; characteristics and contraceptive care experiences	None	Primary	73% prefer obtaining contraception from more than one source. 25% preferred in-person and 19% preferred offsite telemedicine providers. Mistrust led to higher levels of offsite preference.
Lindberg *et al*^51^	6211	Contraceptive services received within 6 months	None	Primary	34% received contraceptive services in 6 months before the survey. 17% used telehealth. Uninsured had great odds of using telehealth (OR 2.93, 95% CI 2.18 to 3.93).
Mastorino *et al*^49^	196	Agreement between triage presumptive diagnosis and medical examination; waiting times	In-person	Primary	Agreement of 74% between telephone and in-person diagnosis. Waiting times not adequate from public health perspective
Merz-Herrala *et al*^64^	2031	Contraceptive appointment attendance; type of appointment; telehealth visit quality	None	Primary	73.4% had an appointment (35.6% telehealth). Telehealth use was less likely in the Midwest and South (aOR 0.63 and 0.54). Lower telehealth quality odds were seen for Hispanic/Latinx and Midwest respondents (aOR 0.37 and 0.58).
Nadarzynski *et al*^37^	257	Acceptability, utilisation and willingness to use of technology; preferred methods of first contact	None	Primary	70% preferred in-person as first point of contact, but 58% willing to have video consultations and 73% use webchats. Several factors associated with acceptability, for example, younger age (<25 years) (OR 2.43, 95% CI 1.35 to 4.38) and White ethnicity (OR 2.87, 95% CI 1.30 to 6.34).
Phillips *et al*^21^	20	Changes in consultations offered; service delivery changes	None	Primary	Clinics increased the proportion of patients having sexual history taken remotely. 75% tried to reduce in-person appointments, 50% using phone appointments. Many clinics did not offer appointments to asymptomatic patients. Phone consultations were used for HIV medication refill.
Player *et al*^38^	20	Patient adherence to visits and medication; patient satisfaction; clinical outcomes.	First e-visit and last e-visit	Primary	75% were comfortable with video calls. At each first 3 month e-visit, medication adherence remained at 68–70%. At the final e-visit, adherence was 60%. 87.5% rated satisfaction as 9 out of 10.
Refugio *et al*^39^	25	Perceptions and experiences of PrEP via Telehealth	None	Primary	At least 75% reported PrEPTECH to be fast, convenient and easy to use. More than 75% said PrEPTECH was very or extremely confidential and would continue using services if they were not free.
Rose *et al*^40^	500	Need for sexual healthcare during lockdown; experiences of telehealth consultations and future access	None	Primary	22% had sexual health needs during lockdown. 40% had an in-person appointment and 60% received telehealth. 46.4% agreed that telehealth was easier than going into clinic. Some dissatisfaction was found with telehealth.
Shanks *et al*^72^	8562 SMS	Volume of SMS text messages; nature of SMS messages sent	None	Secondary	5447 (63.6%) administrative and 3115 (36.4%) clinical SMS messages were received. Reasons for messaging included help with the self-testing process and clinical advice.
Stifani *et al*^48^	172	Prior and current experience with telemedicine; referral patterns; preferences	None	Primary	80% of providers agreed that telemedicine is effective for contraception counselling. 64% of providers would be happy for telemedicine to become a routine part of clinical practice post COVID-19. 60% preferred video visits over phone visits.
Yarger *et al*^41^	1414	Perceived difficulty having a telemedicine visit for contraception.	Food and housing insecurity	Primary	29% food insecure and 15% housing insecure. Food insecure (aOR 2.17; 95% CI 1.62 to 2.91) and housing insecure (aOR 1.62; 95% CI 1.13 to 2.33) participants were significantly more likely to report difficulty with telemedicine for contraception during the pandemic.
Zapata *et al*^29^	1063	Family planning clinical services provided; strategies adopted during COVID-19	None	Primary	Physicians reported experiencing challenges with telehealth services at any point during the pandemic compared in the month before survey completion: technical challenges (45.8% vs 31.7%); confidentiality concerns (21.8% vs 17.0%); billing challenges (32.7% vs 23.1%); and patient discomfort (31.2% vs 21.9%).

* 3338 consultations pre-period (2011–2012) and 3942 post-period (2013–2014) equals a total of 7280 total consultations.

† 197 patients with chlamydia were recruited from GUM clinics and 146 from the NCSP Checkurself service.

‡ *The EmERGE pathway was developed as part of the 'Evaluating mHealth technology in HIV to improve Empowerment and healthcare utilization: Research and Innovation to Generate Evidence for Personalized Care* (EmERGE) Project'

aOR, adjusted OR; GUM, genitourinary medicine; NCSP, National Chlamydia Screening Programme; PrEP, pre-exposure prophylaxis; SRH, sexual and reproductive health; SRHS, sexual and reproductive health services; STIs, sexually transmitted infections.

**Table 3:** Main findings from the mixed methods studies on remote consultations in SRHS

Study	Study design and methods	Stage of integration	Outcome measures	Sample size	Main findings
Auchus *et al*^19^	Concurrent parallel; Q	Interpretation	Appointment attendance rates; patient perceptions of telehealth	4693 unique appointments 202 patient responses	Total clinic non-attendance rates: pre-COVID=15.6% vs post-COVID=12.7% (p=0.01). 46.9% preferred in-person, 11.3% preferred telehealth and 41.8% liked both. Advantages: convenience, safety and privacy benefits. Disadvantages: technical issues, unfamiliarity, lack of human contact and connection and communication.
Giorlando *et al*^57^	Sequential explanatory; Q and I	Interpretation	Comfort levels receiving PrEP at eight locations	QN: 63 QL: 26	Mail delivery (mean=5.14) and telemedicine (mean=4.89) were most comfortable for receiving PrEP. Gyms were the least comfortable (mean=3.92). Women were less comfortable than men receiving PrEP at the locations (mean=4.08 vs 5.07). Advantages: time-saving, convenient, comfortable, enhanced privacy and easy to navigate. Disadvantages: May exclude people without smart devices or wireless connection, delay receiving results and no access to a private area at home or work
Golub *et al*^73^	Embedded; IST and I	Interpretation	Feasibility, acceptability and impact of expanding HIV partner services model	QN: 38 QL: 9	The pilot studies found the programme to be feasible. Participants reported a high level of acceptability and satisfaction.
Lunt *et al*^36^	Sequential explanatory; Q and I	Interpretation	Perceived effectiveness of digital and remote communication channels	QN: 177 QL: 24	Telephone and email were the main communication channels, but perceived effectiveness varied (94% and 66%). Providers had positive attitudes towards digitalisation but had concerns about worsening health inequalities.
Zimbile *et al*^55^	Sequential explanatory; Q, I and FGD	Interpretation	Consultation preferences and quality of care	433 young clients 9 nurses	Video and in-person consultations were positively assessed, and satisfaction was high for both. Advantages for video: accessibility, quicker, timesaving, comfortable, increased anonymity and reduced travel time. Disadvantages for video: technical issues, poor at home testing instructions, lack of eye contact and non-verbal communication.

FGD, focus group discussions; I, interviews; IST, implementation science trial; PrEP, pre-exposure prophylaxis; Q, questionnaires/surveys; QL, qualitative; QN, quantitative; SRHS, sexual and reproductive health services.

**Table 4:** Main findings from the qualitative studies on remote consultations in SRHS

Study	Sample size	Methods	Key areas of interest	Main findings	Example quotes
Aicken *et al*^56^	25	I; TA	E-health acceptability, STI self-testing	Increased convenience and anonymity online, but privacy and confidentiality and anxiety around presence of app for STI testing on phone. Concerns about the accuracy of self-testing. NHS association increased trust in the online pathway. Trade-offs between speed and privacy, and speed and perceived accuracy.	“You could be in the bath, be like using the toilet, and be like, let me just get this real quick and do this real quick. It’s… convenient, very convenient. That’s why I like it” (V, young man, 18–19 years old)
Aicken *et al*^62^	36	I; TA	E-health usage	Privacy using E-services generally viewed as an advantage. Data security concerns and some difficulties when collecting treatment from pharmacy. Speed was key for young people. Trust was linked to the NHS association.	“I knew that the (home sampling-)kit was from the NHS. I, I just trusted everything that came with it, so I trusted the text, the link, and my results. I also trusted the treatment.” (21-year-old woman, tested via Checkurself)
Baraitser and Lupton^61^	10	I; TDA	Digitised SRH diagnosis	Digitised SRH allows anonymous encounters. Experiences of technical challenges with photos and user error. Most participants preferred in-person care. NHS logo increases trust.	“I think it’s a very scary feeling just to put some irritated genitals on the internet and hope for the best … I did have trust and confidence in the service because there were follow-up messages. So there’s those follow-up texts, ‘How is everything working?’” (participant 2)
Bittleston *et al*^52^	114	Q; CA	Telehealth acceptability	Telehealth is convenient and easy to navigate. Technical difficulties, privacy concerns, billing issues and lack of reliable internet. Appropriate for ongoing or uncomplicated issues, for example, repeat prescriptions and routine STI testing.	“Telehealth has been very helpful for repeating scripts and scripts for common infection such as UTI. I hope it continues post COVID-19 into the future” (25/Female/Vic./S3)
Bosó Pérez *et al*^58^	20	I; TA	Pandemic SRH access	Increased convenience via Telehealth. Some contradictory information was given, privacy concerns and difficulty navigating services alone Limits to the suitability of telemedicine. Cultural factors influencing perceptions of remote care.	“I find them [telemedicine appointments] less personal … when you’re on the phone it’s sort of not as easy to remember what you wanted to say, or what you need to ask.” (F, 30–39)
Huang *et al*^42^	44	I; TA and CA	Telehealth implementation	Telehealth is convenient and efficient. Concerns about privacy issues and difficulty communicating complex needs.	“When you’re on a telehealth visit, like a video or telephone, you’re not sure exactly where that patient is, who else like, can hear what they’re saying. So I think it took me a second to figure, you know how I was going to approach that.”
Rao *et al*^43^	546	Q; CA	Telehealth experiences	Telehealth ensured continuity of care, removed physical barriers and enabled some patients to be more willing to share. Although the inability to provide a full contraception range less personal connection, lack of visual aids and digital literacy issues.	"Sometimes people are more willing to share over the phone ambiguity makes them feel safer when they do not have to have a face-to-face interaction with a clinician.” (registered nurse, college health centre, South Carolina)
Ryu *et al*^44^	18	I; GT	COVID-19 testing impact	Remote consultations are highly acceptable and positively viewed by staff and clients, efficient, cost-effective and less onerous. Issues of forging meaningful connections via virtual clinic. Issues of privacy at home and disclosure. Pandemic allowed innovations in service delivery to happen.	“(With virtual services) it’s meant less opportunities for additional education. It’s meant a lot of clients are facing challenges around having a confidential space to have their appointments in, having time set aside (…) so like they might be taking their call on their break from work. They might be—I’ve talked to a bunch of people who are walking outside to take the call because otherwise they would be at home with their parents.” (individual interview)
Sullivan *et al*^59^	4*	I and W; FA	Online safeguarding disclosure	Online services may be more convenient and familiar than a clinic setting. May avoid the embarrassment of discussing sensitive issues in person. Concerns about data sharing and inadvertent breaches of their confidentiality from online service use via visible internet history or testing kits arrived at home.	“I feel quite strongly that there’s no point in saying ‘this is a confidential service.’ I think we have to say, ‘I can’t promise absolute confidentiality as I don’t know what you’re going to tell me. But if you tell me something that raises concern, I might need to share that info.” Professional
Sundstrom *et al*^54^	52	I; GT	Access and rural telehealth	Videoconferencing is a convenient and acceptable way to access contraception. Reduced travel distances and costs, for example, gas/transport. Confidentiality concerns, patient–provider relationship concerns may not facilitate relationship building. Majority of participants expressed positive attitudes towards telehealth for contraception.	“The closer the better because especially from where we live, gas and transportation is always an issue. So, having some place closer would be great.”
Üsküp *et al*^45^	23	FGD/W; TA	Technology acceptability and PrEP for BLCW	Digital technologies can increase flexibility, convenience and remove transport barriers. Offer improved access and communication to service providers. Barriers are out-of-pocket costs not covered by insurance, for example, consultation fees, lab work, prescriptions. Home delivery not always suitable. Impact on undocumented women. Virtual visits may be too impersonal. Customising to meet the needs of the client. BLCW should be able to connect virtually to other women, for example, online chat or community forums.	“Where do transient people go to have medications delivered that is accessible within a space, [and] that is available to them at their leisure and convenience?”
Wells *et al*^53^	45†	I; TA	HIV results delivery	Telephone calls asking for patients to return to clinic cause anxiety. Telephone delivery is time-saving and increases accessibility. However, loses visual cues. For providers, telephone is a less preferable option to convey HIV diagnosis.	“(I’d)had the experience of getting gonorrhoea [previously] so I knew that they text you what you have. And since they didn’t tell me, I was super nervous.”
Yelverton *et al*^46^	11	I; TA	HIV telehealth utilisation	Telehealth frequency, including telephone, video, apps and others rapidly expanded during COVID-19. Barriers: Technology access and security issues, lack of familiarity or comfort and bureaucratic and reimbursement issues. Strategies to improve telehealth: Staff education and training, client empowerment and technology use guidance and bureaucracy and process adjustments.	“when I do like the nurse intake part of that telehealth appointment, I also go through and I try to call them at least an hour before their scheduled time so that we can troubleshoot as needed.”…“in between our EMR team and our IT team, they’ve developed little videos, little things on our website that walk a patient right through step by step on what to do and how to do it.” (interview #5).
Zhang *et al*^47^	47	I; TA and CA	Telemedicine experience	Telemedicine is convenient, reduces physical barriers, although some service users prefer discussing sensitive issues at home. Privacy, cybersecurity and confidentiality, delayed diagnosis and reimbursement issues were all concerns raised. Younger patients were more likely to endorse telemedicine. Patient acceptance varied by experience.	“my phone wasn’t really equipped to do that, so I don’t think on my end that it was not very good ‘cause my phone was very, um, glitchy. My device, I didn’t trust it. My phone wasn’t really equipped to handle that. It’s very horrible to hear, couldn’t really see her too well.” (woman #6, 36 years)

* Four interviews and a workshop of seven; however, it is unclear how the workshop is integrated into the study.

† A total of 35 individuals recently diagnosed with HIV participated completed 59 interviews in total, as some participants had a follow-up interview.

BLCW, black Latina cisgender women; CA, content analysis; EMR, electronic medical record; FA, framework approach; FGD, focus group discussions; GT, grounded theory; I, interviews; IT, information technology; NHS, National Health Service; PrEP, pre-exposure prophylaxis; Q, questionnaires; SRH, sexual and reproductive health ; SRHS, sexual and reproductive health services; STI, sexually transmitted infection; TA, thematic analysis; TDA, theory-driven approach; UTI, urinary tract infection; W, workshops.

### Evidence on clinical effectiveness and cost-effectiveness

A range of outcome measures were used to measure clinical effectiveness reflecting specific populations or disease areas. Examples included attendance rates,^19^,^21^ access to testing and results,^20 22^ completion of telehealth visits,^23^ access to follow-up care,^24^ CD4 cell count,^25 26^ collection, possession of medication and treatment^27 28^ and long-acting reversible contraception (LARC) placement/removal.^29^ Several studies also examined patient views and satisfaction with telehealth services.^1930^,^41^ Most of the outcome measures were intermediate endpoints, and longer-term effectiveness was not analysed. Quality of life was only assessed in both studies by Beck *et al*.^25 26^ Some studies assessed provider provision of and views on telehealth.^2122 29 36 42^,^48^

For the studies that included clinical outcomes, most found that there was no difference in the effectiveness of services provided remotely versus in-person over the period considered. For example, Beck *et al*^25^ found no significant differences in median CD4 count following the introduction of a mobile app to enable people living with HIV to communicate with caregivers. Greenwell *et al*^27^ found no significant difference in PrEP prescriptions filled per person year, between a telehealth clinic and an in-person clinic. Mastorino *et al*^49^ found 73.79% agreement between telephone provisional diagnosis and confirmed syndromic diagnosis, which was deemed to be clinically acceptable.

Most studies that included measures of access or patient perspectives reported no significant differences when comparing method of access, with patient views generally being positive. For example, Bissessor *et al*^20^ found that there was a modest increase in men having HIV tests following provision of test results by phone compared with having to attend in person. A few studies considered access and experiences across different population groups and results were mixed. For example, Ennis *et al*^23^ found that patients with detectable viral load, heterosexuality and older age all had lower odds of completing video telehealth visits over the study period. Hill *et al*^50^ reported that fewer black/African American, compared with other racial and ethnic groups, patients adopted telehealth services compared with in-clinic care. However, Lindberg *et al*^51^ concluded that those from lower socioeconomic groups, those who described themselves as non-Hispanic black, Hispanic or non-Hispanic Asian/Pacific Islander were more likely to report using telehealth.

Very few studies considered costs and cost-effectiveness (table 2). Studies by Beck *et al*^25 26^ reported conflicting findings on the annual costs of virtual HIV clinics: the study from Spain observed an increase in annual costs, while the study from England found a decrease. Bissessor *et al*^20^ examined the time spent on consultations, uptake and attendance and concluded that there were potential savings associated with telephone services, though full costing analysis was not undertaken. Üsküp *et al*^45^ conducted a qualitative study and found that participants believed that telemedicine would be time saving but had concerns about potential out-of-pocket costs. Zapata *et al*^29^ reported that providers had concerns around billing for telehealth.

### Evidence on experiences and access

In the implementation of remote consultations, there were several factors highlighted to ensure acceptability for both service users and service providers. Specific scenarios were identified as suitable for remote consultations, including uncomplicated or ongoing issues such as repeat prescriptions and routine STI testing.^52^ Conway *et al*^31^ found that service users were happy to receive negative HIV test results and post-test discussions over the phone. Wells *et al*^53^ found that healthcare providers were also willing to deliver positive HIV test results remotely, but in-person consultations were preferred because telephone consultations limited the ability in ensuring the emotional well-being of patients. Studies reported that remote consultations could increase access to SRH care^45 48^ by reducing barriers such as distance and access to transport,^4243 45 47 54^,^57^ and cost including childcare and costs of travel.^58^

Remote consultations were found to be acceptable,^44 54^ increased service user convenience,^4547 48 54 56^,^58^ saved time for service users^45 55^ and gave service users more control.^56 59^ Two studies found remote consultations enabled service providers to deliver additional contraception counselling and increase patient knowledge.^43 54^ Johnson^60^ found that women who use contraceptives are more likely to use telemedicine services if they feel comfortable with them and have positive views on telemedicine and direct-to-consumer advertising. Several studies found that implementing remote consultations in SRHS enabled service users to be more open and willing to share information.^43 47 55 61^ Remote consultations could enhance anonymity for service users^48 55 61^ and help avoid embarrassment when discussing sensitive issues.^59^ One study noted that female interviewees particularly valued the increased anonymity of remote consultations.^62^

However, Rao *et al*^43^ found remote consultations created challenges in delivering a full range of contraceptive care, such as LARC placement/removal. Remote consultations also limited the ability to monitor service users with blood pressure checks and physical examinations.^48 58^ There were concerns that remote consultations prevent or delay access to laboratory services and results, causing delayed diagnosis and management.^46 47 57^ Remote consultations reportedly made it more difficult for providers to build rapport and create meaningful connections, as they limited non-verbal cues and eye contact.^44 47 52 55^ This could also create safeguarding challenges.^53^ Some service users noted that remote consultations felt rushed^58^ and inhibited spontaneous discussion.^52^ Several studies reported practical coordination problems when using remote consultations in SRHS, for example, providers finding it difficult to contact service users, patients missing call-backs, challenges in maintaining clinic schedules and difficulty balancing telehealth with in-person visits.^42 46 48 56 58^ Several studies highlighted privacy concerns for both service users and providers,^42 52 54 58^ such as the potential for patients to inadvertently compromise their privacy,^61^ and a lack of privacy at home leading to concerns about disclosure and limiting access.^44 45 57^ Technical problems and internet connectivity issues were found to potentially impact the delivery of remote consultations and the quality of communication.^42 48 52 55^

### The role of remote consultation in reducing or increasing health inequities

Most studies did not directly investigate equity, nor report subgroup findings or disparities. Qualitative studies occasionally discussed equity but rarely as a central focus. Despite this, some studies identified inequities related to geography, age, gender, ethnicity, housing, mental health and substance misuse.

Many of the studies suggested that those living in rural settings face additional barriers to remote access to SRHs.^32 51 53 54 57^ For example, a US study, focussing on remote consultation in HIV services, identified lower access to smartphones and digital technology skills in rural communities.^46^ Age was reported to affect the level of digital skill and/or access to technology, both for older and very young service users.^32 52^ Ethnicity was an important variable, found to influence levels of access to technology and equipment,^42 51^ and exacerbate language and cultural barriers in the remote consultation encounter.^32 58^

Although gender comparisons were limited, studies involving female service users pointed to issues related to reduced digital literacy, limited technology access and privacy concerns.^33 47^ Sexual identity was not found to be a significant barrier to remote consultations, as one study indicated that lesbian, gay and bisexual individuals often preferred remote models.^35^ Similarly, an Australian survey study^33^ found that people with same-sex partners were more willing to have ‘webcam consultations’ for SRHS. Housing insecurity and/or transience were reported as barriers to remote consultations by a small number of studies.^41 45^ In terms of mental health and substance misuse, two studies reported safety concerns from professionals about the risks of interacting via telephone.^36 53^ However, in a US cross-sectional study, Gluskin *et al*^34^ found that ‘videoconferencing may be an acceptable option’ for SRH risk-reduction counselling irrespective of mental health.

Participants in many of the studies had multiple characteristics that could potentially be sources of deprivation or marginalisation, although none formally investigated the intersection between these.^36 41 45 56^ Other elements of intersectionality can be inferred, such as socioeconomic status, which clearly intersected with many of the factors and barriers identified above, including, for example, lack of privacy,^51^ lack of health insurance,^32^ housing insecurity^22^ and ethnicity.^42^ Overall, although intersectionality was rarely formally explored, its relevance to the experience of remote consultation can be inferred, and its importance to future research was acknowledged by some study authors.^54^

### Quality

Quality assessments were performed on all 48 studies using the MMAT, which found that many of the studies did not meet the criteria for a high-quality study (online supplemental file 2 tables 2–5). The qualitative studies included in the review were generally found to be of higher quality compared with studies of other designs, and in particular, all studies that used mixed methods or multi-methods failed to meet nearly all the criteria for being of high quality. Some quantitative studies did not report a comparator^32 34^ and many studies acknowledged issues around generalisability,^22 24 31 32 34 35 37 38 40 48^ sample size and composition^1922 27 28 30 33 34 36 38^,^41 48 50 51 63 64^ and short time horizons.^19^ Many acknowledged their limitations around the self-reporting methods adopted.^21 32^ Additionally, there was reliance on hypothetical scenarios in some studies, and on specific experiences during the COVID-19 pandemic in others, and it was acknowledged that these may differ from actual experience in non-pandemic times.^29 33^

## Discussion

This systematic review synthesised evidence from 48 studies on remote consultations in SRHS, exploring their role in effectiveness, cost-effectiveness, experiences, access and equity. The studies were diverse, and outcome measures included rates of attendance, testing, consultation completion and patient satisfaction. Most of the evidence identified had significant limitations, particularly regarding the generalisability of findings beyond the specific study contexts. Additionally, some quantitative studies lacked a comparator, specifically comparing outcomes between in-person and remote consultations, making it difficult to evaluate the relative effectiveness of remote consultations in SRHS. Another limitation was the absence of long-term analysis, as many studies were conducted over just a few months, often during or immediately after the COVID-19 pandemic. Given the marked changes in the delivery of SRHS during and following the pandemic, the existing evidence does not fully explore the long-term effects of remote consultations as they are now delivered.

Patient feedback on telehealth acceptability and access was generally positive,^44 54^ although many studies relied on hypothetical scenarios rather than users attending real services. As a result, the reported views on remote consultations may shift with first-hand experience. Several studies touched on issues of equity, though this was seldom a primary focus. Identified inequities included challenges in access, such as lack of access to devices and/or the internet,^44^ and language barriers.^48^ Language barriers and other vulnerabilities could also be exacerbated by remote interactions. Remote consultations can reduce physical access barriers and offer greater convenience for users. However, challenges with implementation, such as LARC delivery or delays in laboratory services, may make them unsuitable for certain sexual health needs. Privacy concerns and technical challenges were frequently mentioned, particularly with reference to data protection, confidentiality and internet connectivity.^47 55 56 59^ Ensuring privacy is critical for certain groups, such as adolescents, marginalised populations, and where safeguarding concerns are particularly prominent. Ensuring effective privacy is important not only for protecting patient information but also for complying with legal requirements and maintaining trust in healthcare systems, especially as digital health services continue to expand.

### Comparison with other literature

Our findings update and confirm those from previous systematic reviews in related areas. For example, patient views about remote consultations are generally positive but with some reservations around confidentiality, management when symptomatic and connectivity.^65^,^67^ As we also identified, several other studies highlighted a potential impact on equity associated with remote consultations.^68^,^71^

### Strengths and weaknesses of this review

This review has several strengths. It involved a thorough search across multiple databases combined with article hand searching. Further, it focuses on remote consultations and the impact of remote provision on inequalities and inequities, areas that are understudied. One of the weaknesses of this review is that many of the included studies lack a direct comparator, such as remote consultations versus in-person consultations, making it unclear whether the observed outcomes are due to remote consultations or other factors. Further, the heterogeneous nature of study designs and reporting made quality assessment and synthesis challenging.

## Conclusion and recommendations

The need for more research about remote consultations was identified by many studies, particularly to evaluate clinical effectiveness and cost-effectiveness and revise policies on remote appointments.^36 39 40 47 48 54^ Updating national guidelines on digital sexual health to reflect technological changes is recommended.^36 47 48 54^ There is a need to integrate and optimise remote consultations in SRHS, including using digital SRH as a supplement to in-person clinical services,^37^ optimising telemedicine programmes^22 30^ and improving telehealth technologies and capacities through adequate resourcing.^42 72^ Clear recommendations relating to privacy, personal health information^25 26 33^ and ensuring secure electronic communication^56 62^ need to be implemented.

Some recommendations for improving equity in the use of remote consultation have been suggested but are currently underdeveloped. These included ‘client and provider empowerment and training’ to increase digital literacy,^53^ and ‘outreach and education’ to ‘build health literacy skills that enable young people to access and understand information about telemedicine’.^41^ Other suggestions included ‘digital telehealth pods in centralised locations’ as a means of overcoming challenges to access to Wi-Fi and equipment,^23^ online peer support groups,^45^ and the integration of language interpretation services into ‘telehealth platforms’.^43^ Finally, some authors have identified a need to focus on and advocate for vulnerable or marginalised groups in future research concerning the provision and access to remote consultations within SRHS.^22 73^

## Supplementary material

10.1136/sextrans-2024-056458 online supplemental file 1

10.1136/sextrans-2024-056458 online supplemental file 2

## Data Availability

All data relevant to the study are included in the article or uploaded as supplementary information.
