# Supplementary material for: Remote consultations in sexual and reproductive health services: a systematic review of evidence on effectiveness, cost-effectiveness, experiences, access and equity
Source: Sex Transm Infect. 2025 Sep 25;102(2):e056458. doi: 10.1136/sextrans-2024-056458 (PMC13018787; doi:10.1136/sextrans-2024-056458)
Supplement: online supplemental file 1 [file sextrans-102-2-s001.docx]

**Supplemental file 1**

Search conducted, July 27^th^ to 28^th^ 2023

<https://ovidsp.ovid.com/ovidweb.cgi?T=JS&NEWS=N&PAGE=main&SHAREDSEARCHID=13evKn5aWD1j3azcr55siPp6ZNFjubOE7jMNWZjFSShGnOMTXrhGkpX7tkioz0pEp>

Database(s): **Ovid MEDLINE(R) ALL**1946 to July 25, 2023- searched July 27,2023
Search Strategy:

| **#** | **Searches** | **Results** |
| --- | --- | --- |
| 1 | Remote consultation$.mp. or exp Remote Consultation/ | 6565 |
| 2 | (consultation$ adj3 remote$).ti,ab. | 992 |
| 3 | (appoint$ adj3 remote$).ti,ab. | 60 |
| 4 | (attend$ adj3 remote$).ti,ab. | 122 |
| 5 | (teleconsultation$ or tele-consultation$).mp. | 2217 |
| 6 | (telehealth or tele-health).mp. | 14197 |
| 7 | (telemedicine or tele-medicine).mp. | 49177 |
| 8 | (telecare or tele-care).mp. | 1008 |
| 9 | Videoconferencing/ or videoconferenc$.mp. or video conferenc$.mp. or Video recording/ or video record$.mp. or videorecord$.mp. or video call$.mp. or videocall$.mp. | 43932 |
| 10 | exp Cell phone/ or cell phone$.mp. or smart phone$.mp. or smartphone$.mp. or mobile phone$.mp. | 46394 |
| 11 | Text Messaging/ or text messag$.mp. or short messaging service$.mp. or SMS.mp. | 14666 |
| 12 | Mobile Applications/ or mobile app$.mp. or app.mp. or apps.mp. | 51603 |
| 13 | (mhealth or m-health or mobile health).mp. | 17933 |
| 14 | Telephone/ or telephone$.mp. | 75324 |
| 15 | ((consultation$ adj3 (video$ or phone$ or telephone$ or mobile$)) or (appointment$ adj3 (video$ or phone$ or telephone$ or mobile$)) or (attend$ adj3 (video$ or phone$ or telephone$ or mobile$))).mp. | 4273 |
| 16 | (health adj3 (video$ or phone$ or telephone$ or mobile$)).mp. | 15795 |
| 17 | (e-health or ehealth or digital health).mp. | 17746 |
| 18 | (e-medicine or emedicine).mp. | 107 |
| 19 | Computer-Assisted Instruction/ or (computer assisted or computer-assisted).mp. or (computer based or computer-based).mp. | 365466 |
| 20 | Internet-Based Intervention/ or ((internet or online or web$ or digital) adj3 intervention$).mp. | 11417 |
| 21 | ((consultation$ adj3 (internet or online or web$ or social media or WhatsApp or digital or virtual)) or (appointment$ adj3 (internet or online or web$ or social media or WhatsApp or digital or virtual)) or (attend$ adj3 (internet or online or web$ or social media or WhatsApp or digital or virtual))).mp. | 2731 |
| 22 | ((health or healthcare) adj3 (internet or online or web$ or social media or WhatsApp or digital or virtual)).mp. | 24360 |
| 23 | Electronic Mail/ or (email or e mail or e-mail or electronic mail).mp. | 20165 |
| 24 | (technolog* adj5 (health or healthcare or medical or medicine or consultation$ or appoint$ or attend$)).mp. | 53601 |
| 25 | 1 or 2 or 3 or 4 or 5 or 6 or 7 or 8 or 9 or 10 or 11 or 12 or 13 or 14 or 15 or 16 or 17 or 18 or 19 or 20 or 21 or 22 or 23 or 24 | 696579 |
| 26 | sexual health/ or sexual health.mp. | 14727 |
| 27 | exp Sexually Transmitted Diseases/ | 408838 |
| 28 | (sexually transm$ and (disease$ or infect$ or disorder$)).mp. | 52682 |
| 29 | (STD or STDs or STI or STIs).mp. | 28347 |
| 30 | exp Chlamydia/ | 16495 |
| 31 | exp Chlamydia Infections/ | 22563 |
| 32 | Chlamydia.mp. | 32603 |
| 33 | Gonorrhea/ or gonorrh?ea.mp. | 19848 |
| 34 | exp syphilis/ | 29657 |
| 35 | (syphilis or treponema pallidum).mp. [mp=title, book title, abstract, original title, name of substance word, subject heading word, floating sub-heading word, keyword heading word, organism supplementary concept word, protocol supplementary concept word, rare disease supplementary concept word, unique identifier, synonyms, population supplementary concept word, anatomy supplementary concept word] | 39683 |
| 36 | exp HIV testing/ | 7638 |
| 37 | (Human Immunodeficiency Virus test$ or HIV test$ or AIDS test$).mp. | 17475 |
| 38 | (Human Immunodeficiency Virus screen$ or HIV screen$).mp. | 2357 |
| 39 | (Vaccin$ adj3 sexual$).mp. | 570 |
| 40 | exp Papillomavirus Vaccines/ | 10219 |
| 41 | (papillomavirus or HPV).mp. | 67797 |
| 42 | Reproductive health/ or reproductive health.mp. | 23245 |
| 43 | exp Reproductive Health Services/ | 44270 |
| 44 | exp Reproductive Medicine/ or reproductive medicine.mp. | 28545 |
| 45 | exp Contraception/ or contracept$.mp. or family planning.mp. or birth control.mp. | 126504 |
| 46 | exp Abortion, Spontaneous/ | 38935 |
| 47 | exp Abortion, Induced/ | 42862 |
| 48 | (abortion or termination).mp. | 160894 |
| 49 | (pregnancy adj3 (terminat$ or abort$ or adolesc$ or teen$ or unwanted or unintended)).mp. | 33664 |
| 50 | pregnancy in adolescence/ or pregnancy, unplanned/ or pregnancy, unwanted/ | 12958 |
| 51 | 26 or 27 or 28 or 29 or 30 or 31 or 32 or 33 or 34 or 35 or 36 or 37 or 39 or 40 or 41 or 42 or 43 or 44 or 45 or 46 or 47 or 48 or 49 or 50 | 815745 |
| 52 | "organisation for economic co-operation and development"/ | 550 |
| 53 | exp australia/ or "australia and new zealand"/ or austria/ or baltic states/ or exp belgium/ or exp canada/ or chile/ or colombia/ or costa rica/ or czech republic/ or denmark/ or estonia/ or europe/ or exp finland/ or exp france/ or exp germany/ or greece/ or hungary/ or iceland/ or ireland/ or israel/ or exp italy/ or japan/ or korea/ or latvia/ or lithuania/ or luxembourg/ or exp mexico/ or netherlands/ or new zealand/ or north america/ or exp norway/ or poland/ or exp portugal/ or scandinavia/ or sweden/ or slovakia/ or slovenia/ or south korea/ or exp spain/ or switzerland/ or exp united kingdom/ or "turkey (republic)"/ or exp united states/ or western europe/ | 3453910 |
| 54 | european union/ | 17705 |
| 55 | developed country/ | 21382 |
| 56 | 52 or 53 or 54 or 55 | 3469951 |
| 57 | 25 and 51 and 56 | 4479 |
| 58 | limit 57 to yr="2011-2024" | 2678 |

https://ovidsp.ovid.com/ovidweb.cgi?T=JS&NEWS=N&PAGE=main&SHAREDSEARCHID=4tw5iEiH9nYmGD9ZyYskBgUuSdyJ5OHHhnhJ53ssyqDqS32SW9aZd4uiW6Uqvs0jy

Database(s): **Embase**1974 to 2023 July 26; Searched July 27,2023
Search Strategy:

| **#** | **Searches** | **Results** |
| --- | --- | --- |
| 1 | Remote consultation$.mp. or Remote Consultation/ | 15365 |
| 2 | (consultation$ adj3 remote$).ti,ab. | 1507 |
| 3 | (appoint$ adj3 remote$).ti,ab. | 127 |
| 4 | (attend$ adj3 remote$).ti,ab. | 198 |
| 5 | (teleconsultation$ or tele-consultation$).mp. | 16108 |
| 6 | (telehealth or tele-health).mp. | 26541 |
| 7 | (telemedicine or tele-medicine).mp. | 53874 |
| 8 | (telecare or tele-care).mp. | 2219 |
| 9 | Videoconferencing/ or videoconferenc$.mp. or video conferenc$.mp. or Video recording/ or video record$.mp. or videorecord$.mp. or video call$.mp. or videocall$.mp. | 133257 |
| 10 | exp Cell phone/ or cell phone$.mp. or smart phone$.mp. or smartphone$.mp. or mobile phone$.mp. | 62465 |
| 11 | Text Messaging/ or text messag$.mp. or short messaging service$.mp. or SMS.mp. | 20825 |
| 12 | Mobile Applications/ or mobile app$.mp. or app.mp. or apps.mp. | 72618 |
| 13 | (mhealth or m-health or mobile health).mp. | 16250 |
| 14 | ((consultation$ adj3 (video$ or phone$ or telephone$ or mobile$)) or (appointment$ adj3 (video$ or phone$ or telephone$ or mobile$)) or (attend$ adj3 (video$ or phone$ or telephone$ or mobile$))).mp. | 7449 |
| 15 | (health adj3 (video$ or phone$ or telephone$ or mobile$)).mp. | 15359 |
| 16 | (e-health or ehealth or digital health).mp. | 19283 |
| 17 | (e-medicine or emedicine).mp. | 174 |
| 18 | Internet-Based Intervention/ or ((internet or online or web$ or digital) adj3 intervention$).mp. | 13755 |
| 19 | ((consultation$ adj3 (internet or online or web$ or social media or WhatsApp or digital or virtual)) or (appointment$ adj3 (internet or online or web$ or social media or WhatsApp or digital or virtual)) or (attend$ adj3 (internet or online or web$ or social media or WhatsApp or digital or virtual))).mp. | 4530 |
| 20 | ((health or healthcare) adj3 (internet or online or web$ or social media or WhatsApp or digital or virtual)).mp. | 27978 |
| 21 | Electronic Mail/ or (email or e mail or e-mail or electronic mail).mp. | 45791 |
| 22 | (technolog* adj5 (health or healthcare or medical or medicine or consultation$ or appoint$ or attend$)).mp. | 102219 |
| 23 | sexual health/ or sexual health.mp. | 29754 |
| 24 | Sexually Transmitted Diseases/ | 22825 |
| 25 | (sexually transm$ and (disease$ or infect$ or disorder$)).mp. | 70642 |
| 26 | (STD or STDs or STI or STIs).mp. | 45690 |
| 27 | Chlamydia/ | 8747 |
| 28 | Chlamydia Infections/ | 720 |
| 29 | Chlamydia.mp. | 43502 |
| 30 | Gonorrhea/ or gonorrh?ea.mp. | 23061 |
| 31 | syphilis/ | 29301 |
| 32 | (syphilis or treponema pallidum).mp. [mp=title, abstract, heading word, drug trade name, original title, device manufacturer, drug manufacturer, device trade name, keyword heading word, floating subheading word, candidate term word] | 40964 |
| 33 | exp HIV testing/ | 15780 |
| 34 | (Human Immunodeficiency Virus test$ or HIV test$ or AIDS test$).mp. | 27091 |
| 35 | (Human Immunodeficiency Virus screen$ or HIV screen$).mp. | 3493 |
| 36 | (Vaccin$ adj3 sexual$).mp. | 722 |
| 37 | exp Papillomavirus Vaccines/ | 2305 |
| 38 | ("human papillomavirus" or HPV).mp. | 84599 |
| 39 | Reproductive health/ or reproductive health.mp. | 35433 |
| 40 | Contraception/ or contracept$.mp. or family planning.mp. or birth control.mp. | 168296 |
| 41 | exp Abortion, Spontaneous/ | 50503 |
| 42 | exp Abortion, Induced/ | 31842 |
| 43 | pregnancy in adolescence/ or pregnancy, unplanned/ or pregnancy, unwanted/ | 19087 |
| 44 | "organisation for economic co-operation and development"/ | 2559 |
| 45 | exp australia/ or "australia and new zealand"/ or austria/ or baltic states/ or exp belgium/ or exp canada/ or chile/ or colombia/ or costa rica/ or czech republic/ or denmark/ or estonia/ or europe/ or exp finland/ or exp france/ or exp germany/ or greece/ or hungary/ or iceland/ or ireland/ or israel/ or exp italy/ or japan/ or korea/ or latvia/ or lithuania/ or luxembourg/ or exp mexico/ or netherlands/ or new zealand/ or north america/ or exp norway/ or poland/ or exp portugal/ or scandinavia/ or sweden/ or slovakia/ or slovenia/ or south korea/ or exp spain/ or switzerland/ or exp united kingdom/ or "turkey (republic)"/ or exp united states/ or western europe/ | 3757634 |
| 46 | european union/ | 31022 |
| 47 | developed country/ | 35412 |
| 48 | 44 or 45 or 46 or 47 | 3790693 |
| 49 | or/1-22 | 493393 |
| 50 | or/23-43 | 525056 |
| 51 | 48 and 49 and 50 | 2812 |
| 52 | limit 51 to yr="2011-2024" | 2373 |

https://ovidsp.ovid.com/ovidweb.cgi?T=JS&NEWS=N&PAGE=main&SHAREDSEARCHID=15yDH4WdwNeciTMTKHiTpe5k1tBiT8bu2ZHYRTWZEHDBhG9DuspKGBfaIuenrpfeX

Database(s): **HMIC Health Management Information Consortium**1979 to May 2023; searched July 27, 2023
Search Strategy:

| **#** | **Searches** | **Results** |
| --- | --- | --- |
| 1 | Remote consultation$.mp. | 56 |
| 2 | (consultation$ adj3 remote$).ti,ab. | 70 |
| 3 | (appoint$ adj3 remote$).ti,ab. | 4 |
| 4 | (attend$ adj3 remote$).ti,ab. | 3 |
| 5 | (teleconsultation$ or tele-consultation$).mp. | 119 |
| 6 | (telehealth or tele-health).mp. | 820 |
| 7 | (telemedicine or tele-medicine).mp. | 1550 |
| 8 | (telecare or tele-care).mp. | 851 |
| 9 | Videoconferencing/ or videoconferenc$.mp. or video conferenc$.mp. or Video recording/ or video record$.mp. or videorecord$.mp. or video call$.mp. or videocall$.mp. | 450 |
| 10 | exp Mobile telephones/ or (mobile telephone$ cell phone$ or smart phone$ or smartphone$ or mobile phone$).mp. | 436 |
| 11 | Text Messaging/ or text messag$.mp. or short messaging service$.mp. or SMS.mp. | 222 |
| 12 | (Mobile app$ or app or apps).mp. | 205 |
| 13 | (mhealth or m-health or mobile health).mp. | 182 |
| 14 | exp Telephone/ or telephone$.mp. [mp=title, other title, abstract, heading words] | 4283 |
| 15 | ((consultation$ adj3 (video$ or phone$ or telephone$ or mobile$)) or (appointment$ adj3 (video$ or phone$ or telephone$ or mobile$)) or (attend$ adj3 (video$ or phone$ or telephone$ or mobile$))).mp. | 646 |
| 16 | ((health or healthcare) adj3 (internet or online or web$ or social media or WhatsApp or digital or virtual)).mp. | 1044 |
| 17 | (e-health or ehealth or digital health).mp. | 404 |
| 18 | (e-medicine or emedicine).mp. | 8 |
| 19 | (nternet-Based Intervention or ((internet or online or web$ or digital) adj3 intervention$)).mp. [mp=title, other title, abstract, heading words] | 145 |
| 20 | ((consultation$ adj3 (internet or online or web$ or social media or WhatsApp or digital or virtual)) or (appointment$ adj3 (internet or online or web$ or social media or WhatsApp or digital or virtual)) or (attend$ adj3 (internet or online or web$ or social media or WhatsApp or digital or virtual))).mp. | 156 |
| 21 | (technolog* adj5 (health or healthcare or medical or medicine or consultation$ or appoint$ or attend$)).mp. | 4358 |
| 22 | sexual health.mp. or exp Sexual health/ | 1475 |
| 23 | Sexually Transmitted Diseases.mp. or exp Sexually transmitted infections/ | 772 |
| 24 | (sexually transm$ and (disease$ or infect$ or disorder$)).mp. | 1060 |
| 25 | (STD or STDs or STI or STIs).mp. | 364 |
| 26 | exp Chlamydia infections/ or Chlamydia.mp. | 367 |
| 27 | exp Gonorrhoea/ or Gonorrhea.mp. | 60 |
| 28 | exp Gonorrhoea/ or Gonorrh?ea.mp. | 120 |
| 29 | exp Syphilis/ | 69 |
| 30 | (syphilis or treponema pallidum).mp. | 139 |
| 31 | exp HIV tests/ | 122 |
| 32 | (Human Immunodeficiency Virus test$ or HIV test$ or AIDS test$).mp. | 395 |
| 33 | (Human Immunodeficiency Virus screen$ or HIV screen$).mp. | 62 |
| 34 | (Vaccin$ adj3 sexual$).mp. | 8 |
| 35 | exp Human papillomavirus/ | 368 |
| 36 | exp Human papillomavirus/ or HPV.mp. | 434 |
| 37 | (human papillomavirus vaccine or HPV vaccine).mp. [mp=title, other title, abstract, heading words] | 141 |
| 38 | Reproductive health.mp. | 251 |
| 39 | Reproductive Health Services.mp. | 34 |
| 40 | exp Contraception/ or contracept$.mp. or exp family planning/ or family plan$.mp. or birth control.mp. | 1786 |
| 41 | (abortion or termination).mp. | 1299 |
| 42 | (pregnancy adj3 (terminat$ or abort$ or adolesc$ or teen$ or unwanted or unintended)).mp. | 700 |
| 43 | (adolescent pregnancy or unplanned pregnancy or unwanted pregnancy).mp. | 91 |
| 44 | or/1-21 | 12090 |
| 45 | or/22-43 | 5880 |
| 46 | 44 and 45 | 147 |
| 47 | limit 46 to yr="2011-2024" | 59 |

<https://ovidsp.ovid.com/ovidweb.cgi?T=JS&NEWS=N&PAGE=main&SHAREDSEARCHID=7aQRyZt177HkWDUUJrMMtIRCE0yzXo29VrhmmfXmfcfiGFmcOkbkDNUGaeIE1HWiK>

Database(s): **APA PsycInfo**1967 to July Week 3 2023, searched July 27, 2023
Search Strategy:

| **#** | **Searches** | **Results** |
| --- | --- | --- |
| 1 | Remote consultation$.mp. | 815 |
| 2 | (consultation$ adj3 remote$).ti,ab. | 100 |
| 3 | (appoint$ adj3 remote$).ti,ab. | 3 |
| 4 | (attend$ adj3 remote$).ti,ab. | 32 |
| 5 | (teleconsultation$ or tele-consultation$).mp. | 307 |
| 6 | (telehealth or tele-health).mp. | 3976 |
| 7 | (telemedicine or tele-medicine).mp. | 10303 |
| 8 | (telecare or tele-care).mp. | 297 |
| 9 | Videoconferencing/ or videoconferenc$.mp. or video conferenc$.mp. or Video recording/ or video record$.mp. or videorecord$.mp. or video call$.mp. or videocall$.mp. | 13947 |
| 10 | cell phone.mp. or exp Mobile Phones/ or mobile phon$.mp. or exp exp Smartphones/ or smart phone.mp. | 11071 |
| 11 | Text Messaging/ or text messag$.mp. or short messaging service$.mp. or SMS.mp. | 5169 |
| 12 | Mobile Applications/ or mobile app$.mp. or app.mp. | 11525 |
| 13 | (mhealth or m-health or mobile health).mp. | 3242 |
| 14 | ((consultation$ adj3 (video$ or phone$ or telephone$ or mobile$)) or (appointment$ adj3 (video$ or phone$ or telephone$ or mobile$)) or (attend$ adj3 (video$ or phone$ or telephone$ or mobile$))).mp. | 1194 |
| 15 | (health adj3 (video$ or phone$ or telephone$ or mobile$)).mp. | 3814 |
| 16 | (e-health or ehealth or digital health).mp. | 3738 |
| 17 | (e-medicine or emedicine).mp. | 7 |
| 18 | (Internet-Based Intervention or ((internet or online or web$ or digital) adj3 intervention$)).mp. | 6627 |
| 19 | ((consultation$ adj3 (internet or online or web$ or social media or WhatsApp or digital or virtual)) or (appointment$ adj3 (internet or online or web$ or social media or WhatsApp or digital or virtual)) or (attend$ adj3 (internet or online or web$ or social media or WhatsApp or digital or virtual))).mp. | 699 |
| 20 | ((health or healthcare) adj3 (internet or online or web$ or social media or WhatsApp or digital or virtual)).mp. | 6638 |
| 21 | (technolog* adj5 (health or healthcare or medical or medicine or consultation$ or appoint$ or attend$)).mp. | 9841 |
| 22 | sexual health/ or sexual health.mp. | 8276 |
| 23 | Sexually Transmitted Diseases/ | 4997 |
| 24 | (sexually transm$ and (disease$ or infect$ or disorder$)).mp. | 10842 |
| 25 | (STD or STDs or STI or STIs).mp. | 7558 |
| 26 | (Chlamydia or Chlamydia Infections).mp. | 1045 |
| 27 | Gonorrhea/ or gonorrh?ea.mp. | 796 |
| 28 | exp Syphilis/ | 608 |
| 29 | (syphilis or treponema pallidum).mp. | 1454 |
| 30 | exp HIV Testing/ | 3183 |
| 31 | (Human Immunodeficiency Virus test$ or HIV test$ or AIDS test$).mp. | 5557 |
| 32 | (Human Immunodeficiency Virus screen$ or HIV screen$).mp. | 392 |
| 33 | (Vaccin$ adj3 sexual$).mp. | 95 |
| 34 | Papillomavirus Vaccines.mp. | 710 |
| 35 | HPV.mp. or exp Human Papillomavirus/ | 2302 |
| 36 | Reproductive health/ or reproductive health.mp. or reproductive medicine.mp. | 7079 |
| 37 | Birth Control/ or contracept$.mp. or Family Planning/ or family planning.mp. | 13802 |
| 38 | (abortion or termination).mp. | 22542 |
| 39 | Adolescent Pregnancy/ or unplanned pregnancy.mp. or unwanted pregnancy.mp. | 4266 |
| 40 | or/1-21 | 67507 |
| 41 | or/22-39 | 64819 |
| 42 | 40 and 41 | 1439 |
| 43 | limit 42 to yr="2011-2024" | 1188 |

**Cochrane Library-** 27/07/2023

| # | Search Query | Hits |
| --- | --- | --- |
| 1 | remote consult* | 1274 |
| 2 | (teleconsultation* OR tele-consultation*):ti,kw | 750 |
| 3 | (telehealth OR tele-health):ti,kw | 2562 |
| 4 | (telemedicine OR tele-medicine):ti,kw | 5024 |
| 5 | (telecare OR tele-care):ti,kw | 168 |
| 6 | (videoconferenc* OR video conferenc* OR video call* OR videocall*):ti,kw | 1457 |
| 7 | ("cell phone" OR "smart phone" OR "smartphone" OR "mobile phone"):ti,kw | 5718 |
| 8 | (text messag* OR short messag* service* OR SMS):ti,kw | 3332 |
| 9 | ((mobile NEXT app*)OR (app* NEAR/3 health*):ti,kw) | 4705 |
| 10 | (mhealth OR m-health OR mobile health):ti,kw | 3913 |
| 11 | (e-health OR ehealth OR digital health):ti,kw | 1671 |
| 12 | e-medicine OR emedicine | 21 |
| 13 | intervention NEAR/3 (internet OR web* OR online):ti,kw | 3119 |
| 14 | consultation* NEAR/3 (internet OR online OR web* OR social media OR WhatsAPP OR digital OR virtual):ti,kw | 46 |
| 15 | appointment* NEAR/3 (internet OR online OR web* OR social media OR WhatsAPP OR digital OR virtual):ti,kw | 9 |
| 16 | attend* NEAR/3 (internet OR online OR web* OR social media OR WhatsAPP OR digital OR virtual):ti,kw | 19 |
| 17 | (email OR e mail OR e-mail OR electronic mail):ti,kw | 1952 |
| 18 | (technolog* NEAR/5 (health* OR medic* OR consultation* OR appoint* OR attend*)):ti,ab,kw | 4784 |
| 19 | {OR #1-#18} | 29912 |
| 20 | (sexual health OR sexually transmitted disease):ti,kw | 4424 |
| 21 | (sexually transmitted infection* OR sexually transmitted disorder*):ti,kw | 1245 |
| 22 | (STD OR STDs OR STI OR STIs):ti,kw | 530 |
| 23 | (Chlamydia OR Chlamydia Infections):ti,kw | 1327 |
| 24 | (Gonorrh?ea):ti,kw | 1083 |
| 25 | Syphilis OR treponema pallidum | 949 |
| 26 | (Human Immunodeficiency Virus test* OR HIV test* ):ti,kw | 3327 |
| 27 | (Human Immunodeficiency Virus screen* OR HIV screen*):ti,kw | 808 |
| 28 | ("human papillomavirus" OR HPV):ti,kw | 2637 |
| 29 | (Reproductive health OR reproductive medicine):ti,kw | 1115 |
| 30 | (contracept* OR family planning):ti,kw | 7686 |
| 31 | (abortion OR termination OR "unwanted pregnancy" OR "unplanned pregnancy" OR adolescen* NEAR/1 pregnancy ):ti,kw | 9352 |
| 32 | {OR #20-#31} | 29351 |
| 33 | #19 AND #32 with Cochrane Library publication date Between Jan 2011 and Dec 2024 | 938 |

**NHS EED (CRD)-** July 27,2023

| # | Search Query | Hits |
| --- | --- | --- |
| 1 | (consultation, remote OR remote consult*) | 32 |
| 2 | (teleconsultation* OR tele-consultation*) | 7 |
| 3 | (telehealth OR tele-health) | 19 |
| 4 | (telemedicine OR tele-medicine) | 140 |
| 5 | (telecare OR tele-care) | 28 |
| 6 | (videoconferenc* OR video conferenc* OR video record* OR videorecord* OR video call* OR videocall*):ti,kw | 23 |
| 7 | (cell phone* OR smart phone* OR smartphone* OR mobile phone* ) | 14 |
| 8 | (text messag* OR short messag* service* OR SMS) | 7 |
| 9 | (mhealth OR m-health OR mobile health) | 16 |
| 10 | (Telephone*) | 582 |
| 11 | (e-health OR ehealth OR digital health) | 27 |
| 12 | e-medicine OR emedicine | 0 |
| 13 | (Internet-Based Intervention) | 1 |
| 14 | (internet OR web* OR online) | 590 |
| 15 | #1 OR #2 OR #3 OR #4 OR #5 OR #6 OR #7 OR #8 OR #9 OR #10 OR #11 OR #12 OR #13 OR #14 | 1268 |
| 16 | (sexual health OR sexually transmitted disease) | 51 |
| 17 | (sexually transm* AND (disease* OR infect* OR disorder*)) | 172 |
| 18 | (STD OR STDs OR STI OR STIs) | 98 |
| 19 | (Chlamydia OR Chlamydia Infections) | 108 |
| 20 | (Gonorrh?ea) | 43 |
| 21 | (Syphilis OR treponema pallidum) | 45 |
| 22 | (Human Immunodeficiency Virus test* OR HIV test* OR AIDS test* ) | 80 |
| 23 | (Human Immunodeficiency Virus screen* OR HIV screen*) | 52 |
| 24 | (papillomavirus OR HPV) | 208 |
| 25 | (Reproductive health OR reproductive medicine) | 22 |
| 26 | (reproductive health services) | 2 |
| 27 | (contracept* OR family planning OR birth control) | 116 |
| 28 | (spontaneous abortion OR induced abortion) | 25 |
| 29 | (abortion OR termination) | 173 |
| 30 | #16 OR #17 OR #18 OR #19 OR #20 OR #21 OR #22 OR #23 OR #24 OR #25 OR #26 OR #27 OR #28 OR #29 | 778 |
| 33 | #15 AND #30 | 63 |
| 34 | ( #15 AND #30) FROM 01/01/2011 TO 01/01/2024 | 24 |

<https://www.webofscience.com/wos/woscc/summary/0331d2e6-0bde-4667-932d-c0ea755e16e8-977a3b4a/relevance/1>

**Web of Science – Social Science Citation Index –** July 27, 2023

| # | Search Query | Hits |
| --- | --- | --- |
| 1 | All= (remote consultation*) Editions: WOS.SSCI | 967 |
| 2 | TS=(appoint* NEAR/3 remote*) Editions: WOS.SSCI | 18 |
| 3 | TS=(attend* NEAR/3 remote*) Editions: WOS.SSCI | 73 |
| 4 | TS=(videoconferenc* OR video conferenc* O OR video call* OR videocall*) Editions: WOS.SSCI | 5127 |
| 5 | TS= (cell phone* OR smart phone* OR smartphone* OR mobile phone*) Editions: WOS.SSCI | 26657 |
| 6 | TS= (text messag* OR short messaging service* OR SMS) Editions: WOS.SSCI | 9221 |
| 7 | TS= (mhealth OR m-health OR mobile health) Editions: WOS.SSCI | 16378 |
| 8 | TS= (e-health OR ehealth OR digital health) Editions: WOS.SSCI | 19746 |
| 9 | TS= (e-medicine OR emedicine) Editions: WOS.SSCI | 38 |
| 10 | TS= (consultation* NEAR/3 internet OR consultation* NEAR/3 online OR consultation* NEAR/3 web* OR consultation* NEAR/3 social media OR consultation* NEAR/3 digital OR consultation* NEAR/3 virtual) Editions: WOS.SSCI | 768 |
| 11 | #10 OR #9 OR #8 OR #7 OR #6 OR #5 OR #4 OR #3 OR #2 OR #1 Editions: WOS.SSCI | 64457 |
| 12 | TS= (Sexual health OR reproductive health) Editions: WOS.SSCI | 70990 |
| 13 | TS= ( (sexual transm* disease* OR sexually transm* infect* OR sexually transm* disorder*)) Editions: WOS.SSCI | 15485 |
| 14 | ALL= (chlamydia*) Editions: WOS.SSCI | 3432 |
| 15 | ALL= (gonorrh$ea) Editions: WOS.SSCI | 2373 |
| 16 | ALL= (syphilis OR treponema pallidum) Editions: WOS.SSCI | 4150 |
| 17 | TS= ("HIV test*" OR "HIV screen*" ) Editions: WOS.SSCI | 7999 |
| 18 | TS= (HPV OR human papillomavirus) Editions: WOS.SSCI | 7399 |
| 19 | TS= (Birth control OR contracept* OR "family planning") Editions: WOS.SSCI | 43800 |
| 20 | TS= (abortion) Editions: WOS.SSCI | 18196 |
| 21 | #12 OR #13 OR #14 OR #15 OR #16 OR #17 OR #18 OR #19 OR #20 Editions: WOS.SSCI | 141568 |
| 22 | #21 AND #11 Editions: WOS.SSCI | 2578 |
| 23 | #21 AND #11 Editions: WOS.SSCI (2011-2023) | 2438 |

**EconLit (EBSCO)-** July 27,2023

| # | Search Query | Hits |
| --- | --- | --- |
| s1 | remote consult* | 4 |
| s2 | teleconsultation* OR tele-consultation* | 2 |
| s3 | telehealth OR tele-health | 30 |
| s4 | telemedicine OR tele-medicine | 55 |
| s5 | telecare OR tele-care | 5 |
| s6 | videoconferenc* OR video conferenc* OR video record* OR videorecord* OR video call* OR videocall* | 128 |
| s7 | cell phone* OR smart phone* OR smartphone* OR mobile phone* | 2016 |
| s8 | text messag* OR short messag* service* OR SMS | 384 |
| s9 | mhealth OR m-health OR mobile health | 96 |
| s10 | e-health OR ehealth OR digital health | 126 |
| s11 | e-medicine OR emedicine | 0 |
| s12 | Internet Based Intervention | 3 |
| s13 | health* N5 internet OR health* N5 web* OR health* N5 online | 198 |
| s14 | sexual health OR sexually transmitted disease | 269 |
| s15 | sexually transm* AND (disease* OR infect* OR disorder*) | 199 |
| s16 | STD OR STDs OR STI OR STIs | 365 |
| s17 | Chlamydia OR Chlamydia Infections | 26 |
| s18 | Gonorrohea OR gonorrhea | 40 |
| s19 | Syphilis OR treponema pallidum | 41 |
| s20 | Human Immunodeficiency Virus test* OR HIV test* OR AIDS test* | 218 |
| s21 | Human Immunodeficiency Virus screen* OR HIV screen* | 12 |
| s22 | papillomavirus OR HPV | 60 |
| s23 | Reproductive health OR reproductive medicine | 621 |
| S24 | reproductive health services or sexual health services | 115 |
| S25 | contracept* OR family planning OR birth control | 29544 |
| S26 | spontaneous abortion OR induced abortion | 82 |
| S27 | abortion or termination of pregnancy or termination or unintended pregnancy or unwanted pregnancy or unplanned pregnancy | 2671 |
| s28 | S1 OR S2 OR S3 OR S4 OR S5 OR S6 OR S7 OR S8 OR S9 OR S10 OR S11 OR S12 OR S13 | 2907 |
| S29 | S14 OR S15 OR S16 OR S17 OR S18 OR S19 OR S20 OR S21 OR S22 OR S23 OR S24 OR S25 OR S26 OR S27 | 32290 |
| S30 | S28 AND S29 | 131 |
| S31 | S28 AND S29 (Published Date: 20110101-20240131) | 111 |

**Health Systems Evidence**- July 27, 2023

| # | Search Query | Hits |
| --- | --- | --- |
| 1 | remote consultation OR telehealth OR Digital health | 289 |
| 2 | Sexual health OR reproductive health OR chlamydia OR gonorrhoea OR gonorrhea OR chlamydia OR HIV OR contraception OR abortion | 368 |
| 3 | (Sexual health OR reproductive health OR chlamydia OR gonorrhoea OR gonorrhea OR chlamydia OR HIV OR contraception OR abortion) AND (remote consultation OR telehealth OR Digital health) | 11 |

**Database of Promoting Health Effectiveness Reviews (DoPHER)**-July 27, 2023

| Set# | Search query | Hits |
| --- | --- | --- |
| 1 | Freetext (All but Authors): "remote consultation" | 0 |
| 2 | Freetext (All but Authors): telehealth | 19 |
| 3 | Freetext (All but Authors): tele-health | 0 |
| 4 | Freetext (All but Authors): telemedicine | 13 |
| 5 | Freetext (All but Authors): tele-medicine | 1 |
| 6 | Freetext (All but Authors): telecare | 1 |
| 7 | Freetext (All but Authors): tele-care | 0 |
| 8 | (Freetext (All but Authors): "video conference" | 0 |
| 9 | Freetext (All but Authors): "video conferencing" | 5 |
| 10 | Freetext (All but Authors): videoconference | 2 |
| 11 | Freetext (All but Authors): videoconferencing | 3 |
| 12 | Freetext (All but Authors): "video call" | 2 |
| 13 | Freetext (All but Authors): "video calling" | 0 |
| 14 | Freetext (All but Authors): "cell phone" | 6 |
| 15 | Freetext (All but Authors): "smart phone" | 4 |
| 16 | Freetext (All but Authors): smartphone | 67 |
| 17 | Freetext (All but Authors): "mobile phone" | 87 |
| 18 | Freetext (All but Authors): "text message" | 40 |
| 19 | Freetext (All but Authors): "text messaging" | 83 |
| 20 | Freetext (All but Authors): "short message service" | 34 |
| 21 | Freetext (All but Authors): "short messaging service" | 4 |
| 22 | Freetext (All but Authors): SMS | 63 |
| 23 | Freetext (All but Authors): "mobile app" | 22 |
| 24 | Freetext (All but Authors): app | 89 |
| 25 | Freetext (All but Authors): mhealth | 104 |
| 26 | Freetext (All but Authors): "m-health " | 6 |
| 27 | Freetext (All but Authors): "mobile health" | 100 |
| 28 | Freetext (All but Authors): "e-health " | 23 |
| 29 | Freetext (All but Authors): "ehealth " | 71 |
| 30 | Freetext (All but Authors): "digital health" | 31 |
| 32 | Freetext (All but Authors): e-medicine | 0 |
| 33 | Freetext (All but Authors): emedicine | 0 |
| 34 | Freetext (Title): "email " | 3 |
|  | Freetext (Title): e-mail | 0 |
| 35 | Freetext (Title): "e mail" | 0 |
| 36 | Freetext (Title): "electronic mail" | 0 |
| 37 | 1 OR 2 OR 3 OR 4 OR 5 OR 6 OR 7 OR 8 OR 9 OR 10 OR 11 OR 12 OR 13 OR 14 OR 15 OR 16 OR 17 OR 18 OR 19 OR 20 OR 21 OR 22 OR 23 OR 24 OR 25 OR 26 OR 27 OR 28 OR 29 OR 30 OR 31 OR 32 OR 33 OR 34 OR 35 OR 36 | 474 |
| 38 | Freetext (All but Authors): "sexual health" | 133 |
| 39 | Freetext (All but Authors): "sexually transmitted disease" | 21 |
| 40 | Freetext (All but Authors): "sexually transmitted infection" | 38 |
| 41 | Freetext (All but Authors): "sexually transmitted disorder" | 0 |
| 42 | Freetext (All but Authors): STD | 38 |
| 43 | Freetext (All but Authors): STDs | 23 |
| 44 | Freetext (All but Authors): STI | 144 |
| 45 | Freetext (All but Authors): STIs | 85 |
| 46 | Freetext (All but Authors): Chlamydia | 37 |
| 47 | Freetext (All but Authors): Chlamydial | 7 |
| 48 | Freetext (All but Authors): gonorrhoea | 13 |
| 49 | Freetext (All but Authors): gonorrhea | 6 |
| 50 | Freetext (All but Authors): Syphilis | 14 |
| 51 | Freetext (All but Authors): "treponema pallidum" | 0 |
| 52 | Freetext (All but Authors): "Human Immunodeficiency Virus " | 66 |
| 53 | Freetext (All but Authors): HIV | 529 |
| 54 | Freetext (All but Authors): AIDS | 196 |
| 55 | Freetext (All but Authors): "human papillomavirus" | 39 |
| 56 | Freetext (All but Authors): HPV | 47 |
| 57 | Freetext (All but Authors): "Reproductive health" | 89 |
| 58 | Freetext (All but Authors): "reproductive medicine" | 0 |
| 59 | Freetext (All but Authors): "Birth control " | 12 |
| 60 | Freetext (All but Authors): "contracept*" | 131 |
| 61 | Freetext (All but Authors): "family planning" | 57 |
| 62 | Freetext (All but Authors): "abort*" | 27 |
| 63 | Freetext (All but Authors): "terminat*" | 10 |
| 64 | Freetext (All but Authors): "unwanted pregnan*" | 8 |
| 65 | Freetext (All but Authors): "unintended pregnan* | 0 |
| 66 | Freetext (All but Authors): "teen* abort*" | 0 |
| 67 | Freetext (All but Authors): "adolescen* pregnan*" | 35 |
| 68 | 38 OR 39 OR 40 OR 41 OR 42 OR 43 OR 44 OR 45 OR 46 OR 47 OR 48 OR 49 OR 50 OR 51 OR 52 OR 53 OR 54 OR 55 OR 56 OR 57 OR 58 OR 59 OR 60 OR 61 OR 62 OR 63 OR 64 OR 65 OR 66 OR 67 | 941 |
| 69 | 37 AND 68 | 70 |

**International HTA database (INAHTA)-** July 27 2023

| Set# | Search query | Hits |
| --- | --- | --- |
| 1 | (remote consult*) | 17 |
| 2 | ((teleconsultation*)[Title] OR (teleconsultation*)[Keywords]) | 2 |
| 3 | ((telehealth OR tele-health)[Title] OR (telehealth OR tele-health)[Keywords]) | 20 |
| 4 | (telemedicine OR tele-medicine)[Title] OR (telemedicine OR tele-medicine)[Keywords] | 53 |
| 5 | ((telecare OR tele-care)[Title] OR (telecare OR tele-care)[Keywords]) | 1 |
| 6 | ((((videoconferenc* OR video conferenc* OR video call* OR videocall*)[Title] OR (videoconferenc* OR video conferenc* OR video call* OR videocall*)[Keywords]))) | 67 |
| 7 | (("cell phone" OR smart phone OR smartphone OR mobile phone)[Title] OR ("cell phone" OR smart phone OR smartphone OR mobile phone)[Keywords]) | 26 |
| 8 | (text messag* OR short messag* service* OR SMS)[Title] | 495 |
| 9 | ((mhealth OR m-health OR mobile health)[Title] | 1154 |
| 10 | (((e-health OR ehealth OR digital health)[Title] | 1209 |
| 11 | (e-medicine OR emedicine)[Title] OR (e-medicine OR emedicine)[Keyword] | 644 |
| 12 | (internet OR web* OR online)[Title] OR (internet OR web* OR online)[Keyword] | 430 |
| 13 | 1 OR 2 OR 3 OR 4 OR 5 OR 6 OR 7 OR 8 OR 9 OR 10 OR 11 OR 12 | 1663 |
| 14 | ((sexual health OR sexually transmitted disease)[Title] OR (sexual health OR sexually transmitted disease)[keyword])) | 3169 |
| 15 | (STD OR STDs OR STI OR STIs)[Title] OR (STD OR STDs OR STI OR STIs)[Keyword] | 15 |
| 16 | (Chlamydia OR Chlamydia Infections)[Title] OR (Chlamydia OR Chlamydia Infections)[Keyword] | 502 |
| 17 | ((Gonorrhoea OR gonorrhea)[Title] OR (Gonorrhoea OR gonorrhea)[Keyword]) | 4 |
| 18 | (Syphilis or treponema pallidum)[Title] OR Syphilis or treponema pallidum)[Keyword]) | 4 |
| 19 | (Human Immunodeficiency Virus test* OR HIV test*)[Title] | 997 |
| 20 | (Human Immunodeficiency Virus screen* OR HIV screen*)[Title] | 984 |
| 21 | (Human papillomavirus OR HPV)[Title] OR (Human papillomavirus OR HPV)[Keyword] | 89 |
| 22 | (Reproductive health OR reproductive medicine)[Title] OR (Reproductive health OR reproductive medicine)[Keyword] | 711 |
| 23 | (contracept* OR birth control OR family planning)[Title] | 197 |
| 24 | (abortion OR termination)[Title] OR (abortion OR termination)[Keyword] | 47 |
| 25 | 14 OR 15 OR 16 OR 17 OR 18 OR 19 OR 20 OR 21 OR 22 OR 23 OR 24 | 5690 |
| 26 | 13 AND 25 | 1727 |
| 27 | 26 FROM 2011 TO 2024 | 897 |

**Applied Social Science Index and Abstract (ASSIA), International Bibiliography of the Social Sciences (IBSS) and Sociological abstract, (Proquest)-** July 28, 2023

| Set# | Searched for | **ASSIA** | **IBSS** | **Sociological Abstracts** |
| --- | --- | --- | --- | --- |
| S1 | noft(consultation, remote OR remote consult*) | 351 | 165 | 63 |
| S2 | title(telehealth OR tele-health) OR abstract(telehealth OR tele-health) | 1294 | 193 | 145 |
| S3 | title(telemedicine OR tele-medicine) OR abstract(telemedicine OR tele-medicine) | 779 | 290 | 146 |
| S4 | title(telecare OR tele-care) OR abstract(telecare OR tele-care) | 214 | 81 | 77 |
| S5 | title(videoconferenc* OR video conferenc* OR video call* OR videocall*) OR abstract(videoconferenc* OR video conferenc* OR video call* OR videocall*) | 1141 | 1234 | 946 |
| S6 | title(cell phone* OR smart phone* OR smartphone* OR mobile phone*) | 1033 | 1740 | 889 |
| S7 | title(text messag* OR short messag* service* OR SMS) | 392 | 327 | 121 |
| S8 | title("mobile app*" OR app* NEAR/3 health*) | 1428 | 988 | 539 |
| S9 | title(mhealth OR m-health OR mobile health) | 571 | 367 | 99 |
| S10 | title(consultation* NEAR/3 (video* OR phone* OR telephone* OR mobile*)) OR abstract(consultation* NEAR/3 (video* OR phone* OR telephone* OR mobile*)) | 469 | 87 | 76 |
| S11 | title(appointment* NEAR/3 (video* OR phone* OR telephone* OR mobile*)) OR abstract(appointment* NEAR/3 (video* OR phone* OR telephone* OR mobile*)) | 80 | 14 | 9 |
| S12 | title(attend* NEAR/3 (video* OR phone* OR telephone* OR mobile*)) OR abstract(attend* NEAR/3 (video* OR phone* OR telephone* OR mobile*)) | 100 | 43 | 48 |
| S13 | title(e-health OR ehealth OR digital health) | 979 | 396 | 201 |
| S14 | noft(e-medicine OR emedicine) | 12 | 10 | 4 |
| S15 | title(intervention NEAR/3 (internet OR online OR web* OR digital)) | 978 | 188 | 106 |
| S16 | title(consultation* NEAR/3 (internet OR online OR web*)) OR abstract(consultation* NEAR/3 (internet OR online OR web*)) | 149 | 144 | 40 |
| S17 | noft((consultation* NEAR/3 social media)) | 5 | 6 | 1 |
| S18 | noft(consultation* NEAR/3 WhatsApp) | 0 | 1 | 0 |
| S19 | noft(consultation* NEAR/3 (digital OR virtual)) | 62 | 24 | 11 |
| S20 | title(appointment* NEAR/3 (internet OR online OR web*))OR abstract(appointment* NEAR/3 (internet OR online OR web*)) | 29 | 12 | 3 |
| S21 | noft(appointment* NEAR/3 social media) | 2 | 3 | 0 |
| S22 | noft(appointment* NEAR/3 WhatsApp) | 0 | 0 | 0 |
| S23 | title(appointment* NEAR/3 (digital OR virtual))OR abstract(appointment* NEAR/3 (digital OR virtual)) | 13 | 1 | 1 |
| S24 | title(attend* NEAR/3 (internet OR online OR web*)) OR abstract(attend* NEAR/3 (internet OR online OR web*)) | 83 | 76 | 61 |
| S25 | title(attend* NEAR/3 social media ) OR abstract(attend* NEAR/3 social media ) | 15 | 34 | 30 |
| S26 | noft(attend* NEAR/3 WhatsApp) | 0 | 0 | 0 |
| S27 | title(attend* NEAR/3 ( digital OR virtual)) OR abstract(attend* NEAR/3 ( digital OR virtual)) | 34 | 43 | 26 |
| S28 | title(technolog* NEAR/5 (health* OR medic* OR consultation* OR appoint* OR attend*)) | 1132 | 1354 | 672 |
| S29 | 1 OR 2 OR 3 OR 43 OR5 OR 6 OR 7 OR 8 OR 9 OR 10 OR 11 OR 12 OR 13 OR 14 OR 15 OR 16 OR 17 OR 18 OR 19 OR 20 OR 21 OR 22 OR 23 OR 24 OR 25 OR 26 OR 27 OR 28 | 10131 | 7472 | 4095 |
| S30 | title(sexual health OR sexually transmitted disease) OR abstract(sexual health OR sexually transmitted disease) | 16362 | 8885 | 11387 |
| S31 | title(sex* transm* infect* OR "sex* transm* disorder*") OR abstract(sex* transm* infect* OR "sex* transm* disorder*") | 4197 | 1675 | 1305 |
| S32 | title(STD OR STDs OR STI OR STIs) OR abstract(STD OR STDs OR STI OR STIs) | 5022 | 2386 | 1820 |
| S33 | title(Chlamydia*) OR abstract(Chlamydia*) | 2132 | 179 | 112 |
| S34 | title(Gonorrh?ea) OR abstract(Gonorrh?ea) | 1290 | 176 | 131 |
| S35 | title(Syphilis or treponema pallidum) OR abstract(Syphilis or treponema pallidum) | 1550 | 542 | 265 |
| S36 | title(Human Immunodeficiency Virus test* OR HIV test*) | 1821 | 1354 | 485 |
| S37 | title(Human Immunodeficiency Virus screen* OR HIV screen*) | 281 | 123 | 26 |
| S38 | title(human papillomavirus or HPV) OR abstract(human papillomavirus or HPV) | 1818 | 473 | 348 |
| S39 | title(Birth control OR contracept*) | 2581 | 3051 | 2629 |
| S40 | title(abortion OR termination) | 2537 | 5400 | 3818 |
| S41 | 30 OR 31 OR 32 OR 33 OR 34 OR 35 OR 36 OR 37 OR 38 OR 39 OR 40 | 29135 | 20740 | 19383 |
| S42 | S29 AND S41 | 322 | 172 | 82 |
| S43 | S29 AND S41 AND pd(20110101-20240101) | 278 | 139 | 61 |
