# Supplementary material for: Remote consultations in sexual and reproductive health services: a systematic review of evidence on effectiveness, cost-effectiveness, experiences, access and equity
Source: Sex Transm Infect. 2025 Sep 25;102(2):e056458. doi: 10.1136/sextrans-2024-056458 (PMC13018787; doi:10.1136/sextrans-2024-056458)
Supplement: online supplemental file 2 [file sextrans-102-2-s002.docx]

**Supplement file 2**

| **Supplementary Table 1. Study inclusion and exclusion criteria** | |
| --- | --- |
| **Stage 1 – update** | |
|  | The study involves remote consultations in SRHS |
| B. | The study involves remote consultations; however, it is unclear if associated with SRHS |
| C. | Unclear if the study falls under A or B, but may be useful for the review |
| D. | The study is concerned with consultations but not clear if relevant to remote consultations |
| E. | The study is not relevant to remote consultations in SRHS |
| F. | Copy/duplicate publication |
| G. | Non-OECD publication |
| H. | Publication predates 01/01/2011 |
| I. | No abstract |
| J. | Abstract not published in English |
| K. | No methods section |
| **Stage 2** | |
| 1. | The study is concerned with inequalities in relation to remote consultations in SRHS |
| 2. | The study is concerned with remote consultations in SRHS (but not specifically inequalities) |
| 3. | Review, guideline or editorial in relation to remote consultation in SRHS |
| 4. | The study evaluates remote consultation, but does not relate to SRHS |
| 5. | Study not published in English |
| 6. | Not relevant to remote consultation in SRHS |
| 7. | Full text is unavailable |
| 8. | Non-OECD publication |
| 9. | Duplicate study |

**Quality assessment**

| **Supplementary Table 2. Quality assessment for quantitative non-randomised studies** | | | | | | | | | | | | | |
| --- | --- | --- | --- | --- | --- | --- | --- | --- | --- | --- | --- | --- | --- |
|  | **Mixed Methods Appraisal Tool (MMAT)** | **Player et al** | **Refugio et al** | **Beck et al (Spain)** | **Beck et al** | **Green-well et al** | **Ennis et al** | **Estcourt et al** | **Hoth et al** | **Golub et al** | **Bryso-n et al** | **Bisses-ssor et al** | **Masto-rino et al** |
| **Q1** | Are there clear research questions? | Y | Y | Y | Y | Y | Y | Y | Y | Y | Y | Y | Y |
| **Q2** | Do the collected data allow to address the research questions? | Y | Y | Y | Y | Y | Y | Y | Y | Y | Y | Y | Y |
| **Q3** | Are the participants representative of the target population? | N | N | N | N | N | N | Y | N | N | Y | N | N |
| **Q4** | Are measurements appropriate regarding both the outcome and intervention (or exposure)? | Y | Y | Y | Y | Y | Y | Y | Y | Y | Y | Y | Y |
| **Q5** | Are there complete outcome data? | N^1^ | N^2^ | Y | Y | Y | Y | Y | N^3^ | Y | Y | Y | Y |
| **Q6** | Are the confounders accounted for in the design and analysis? | N | N | Y | Y | N | Y | Y | N | N | N | N | N |
| **Q7** | During the study period, is the intervention administered (or exposure occurred) as intended? | Y | Y | N | Y | Y | Y | Y | Y | Y | Y | Y | Y |
| ^1^20 joined, 16 finished the programme  ^2^25 completed baseline and 30-day telehealth visit, 21 completed study. 19% drop out rate over 180 days  ^3^6-month retention was 77% (50 of 65) | | | | | | | | | | | | | |

|  | **Supplementary Table 3. Quality assessment for quantitative descriptive studies** | | | | | | | | | | | |
| --- | --- | --- | --- | --- | --- | --- | --- | --- | --- | --- | --- | --- |
|  | | **Mixed Methods Appraisal Tool (MMAT)** | **Merz- Herrala et al** | **Nadarz-ynski et al** | **Phillips**  **et al** | **Rose et al** | **Yarger et al** | **Zapata et al** | **Johnson** | **Kavana-ugh et al** | **Shanks et al** |  |
| **Q1** | | Are there clear research questions? | Y | Y | Y | Y | Y | Y | Y | Y | Y |  |
| **Q2** | | Do the collected data allow to address the research questions? | Y | Y | Y | Y | Y | Y | Y | Y | Y |  |
| **Q3** | | Is the sampling strategy relevant to address the research question? | Y | Y | Y | Y | N | N | Y | Y | Y |  |
| **Q4** | | Is the sample representative of the target population? | N | N | N | Y | N | Y | N | Y | Y |  |
| **Q5** | | Are the measurements appropriate? | Y | N | N | Y | Y | Y | Y | Y | Y |  |
| **Q6** | | Is the risk of non-response bias low? | N | N | N | N | N | N | N | Y | N |  |
| **Q7** | | Is the statistical analysis appropriate to answer the research question? | Y | Y | N | N | Y | Y | N | Y | Y |  |
|  | |  | **Clure et al** | **Comfort et al** | **Conway et al** | **Garrett et al** | **Gluskin et al** | **Hill et al** | **Lindberg et al** | **Galaviz et al** | **Stifani et al** |  |
| **Q1** | | Are there clear research questions? | Y | Y | Y | Y | Y | Y | Y | Y | Y |  |
| **Q2** | | Do the collected data allow to address the research questions? | Y | Y | Y | Y | Y | Y | Y | Y | Y |  |
| **Q3** | | Is the sampling strategy relevant to address the research question? | Y | Y | N | Y | N | N | Y | Y | Y |  |
| **1** | | Is the sample representative of the target population? | Y | N | Y | N | N | N | N | N | Y |  |
| **Q5** | | Are the measurements appropriate? | Y | Y | N | Y | Y | Y | Y | Y | Y |  |
| **Q6** | | Is the risk of non-response bias low? | N | N | N | N | N | Y | N | N | N |  |
| **Q7** | | Is the statistical analysis appropriate to answer the research question? | Y | Y | Y | Y | Y | Y | Y | Y | Y |  |

| **Supplementary Table 4. Quality assessment for qualitative studies** | | | | | | | | | | | | | | | |
| --- | --- | --- | --- | --- | --- | --- | --- | --- | --- | --- | --- | --- | --- | --- | --- |
|  | **Mixed Methods Appraisal Tool (MMAT)** | **Rao et al** | **Ryu et al** | **Sundst-rom et al** | **Üsküp et al** | **Wells et al** | **Yelver-ton et al** | **Zhang et al** | **Sulliv-an et al** | **Aicken et al^1^** | **Aicken et al^2^** | **Barais-ter et al** | **Bittle-son et al** | **Huang et al** | **Boso Perez et al** |
| **Q1** | Are there clear research questions? | Y | Y | Y | Y | Y | Y | Y | Y | Y | Y | Y | Y | Y | Y |
| **Q2** | Do the collected data allow to address the research questions? | Y | Y | Y | Y | Y | Y | Y | Y | Y | Y | Y | Y | Y | Y |
| **Q3** | Is the qualitative approach appropriate to answer the research question? | Y | Y | Y | Y | Y | Y | Y | Y | Y | Y | Y | Y | Y | Y |
| **Q4** | Are the qualitative data collection methods adequate to address the research question? | N | Y | Y | N | Y | Y | Y | Y | Y | Y | Y | N | Y | Y |
| **Q5** | Are the findings adequately derived from the data? | Y | Y | Y | Y | Y | Y | N | Y | Y | Y | Y | N | Y | Y |
| **Q6** | Is the interpretation of results sufficiently substantiated by data? | Y | Y | Y | Y | Y | Y | N | Y | Y | Y | Y | Y | Y | Y |
| **Q7** | Is there coherence between qualitative data sources, collection, analysis and interpretation? | Y | Y | Y | U | Y | Y | N | N | Y | Y | Y | Y | Y | Y |

| **Supplementary Table 5. Quality assessment for mixed-method studies** | | | | | | |
| --- | --- | --- | --- | --- | --- | --- |
|  | **Mixed Methods Appraisal Tool (MMAT)** | **Lunt et al** | **Zimbile et al** | **Auchus et al** | **Giorlando et al** | **Golub et al** |
| **Q1** | Are there clear research questions? | N | Y | Y | Y | Y |
| **Q2** | Do the collected data allow to address the research questions? | U | Y | Y | Y | U |
| **Q3** | Is there an adequate rationale for using a mixed methods design to address the research question? | U | N | Y | U | U |
| **Q4** | Are the different components of the study effectively integrated to answer the research question? | Y | N | N | U | U |
| **Q5** | Are the outputs of the integration of qualitative and quantitative components adequately interpreted? | U | U | N | U | U |
| **Q6** | Are divergences and inconsistencies between quantitative and qualitative results adequately addressed? | N | U | N | U | U |
| **Q7** | Do the different components of the study adhere to the quality criteria of each tradition of the methods involved? | Y | U | N | U | U |
